# Directionality of Macrophages Movement in Tumour Invasion: A Multiscale Moving-Boundary Approach

**DOI:** 10.1007/s11538-020-00819-7

**Published:** 2020-11-19

**Authors:** Szabolcs Suveges, Raluca Eftimie, Dumitru Trucu

**Affiliations:** Division of Mathematics, University of Dundee, Dundee, DD1 4HN UK

**Keywords:** Cancer invasion, Macrophages, Multiscale modelling, Computational modelling, Cell adhesion, Flux limiter, Convolution

## Abstract

Invasion of the surrounding tissue is one of the recognised hallmarks of cancer (Hanahan and Weinberg in Cell 100: 57–70, 2000. 10.1016/S0092-8674(00)81683-9), which is accomplished through a complex heterotypic multiscale dynamics involving tissue-scale random and directed movement of the population of both cancer cells and other accompanying cells (including here, the family of tumour-associated macrophages) as well as the emerging cell-scale activity of both the matrix-degrading enzymes and the rearrangement of the cell-scale constituents of the extracellular matrix (ECM) fibres. The involved processes include not only the presence of cell proliferation and cell adhesion (to other cells and to the extracellular matrix), but also the secretion of matrix-degrading enzymes. This is as a result of cancer cells as well as macrophages, which are one of the most abundant types of immune cells in the tumour micro-environment. In large tumours, these tumour-associated macrophages (TAMs) have a tumour-promoting phenotype, contributing to tumour proliferation and spread. In this paper, we extend a previous multiscale moving-boundary mathematical model for cancer invasion, by considering also the multiscale effects of TAMs, with special focus on the influence that their directional movement exerts on the overall tumour progression. Numerical investigation of this new model shows the importance of the interactions between pro-tumour TAMs and the fibrous ECM, highlighting the impact of the fibres on the spatial structure of solid tumour.

## Introduction

One of the key factors that distinguish cancer cells from normal cells is the ability of cancer cells to alter their local and non-local interactions to neighbouring cells (that are either cancerous or of different types) and to the extracellular matrix (ECM), which leads eventually to invasion and metastasis (Hanahan and Weinberg 2000, 2011). The ECM consists of a network of macromolecules (i.e. fibrous proteins, water, minerals, proteoglycans), which is present in all tissues and regulates cell behaviour and tissue homeostasis (Filipe et al. 2018). While the structure of the matrix undergoes constant remodelling (via synthesis and degradation), the ECM loses its integrity during cancer progression. There are many enzymes inside the solid tumours that can degrade the ECM, such as matrix metalloproteinases (MMPs) or urokinase plasminogen activator system (uPA), but the complete details of the cell types within the tumour micro-environment that are dominant in secreting these enzymes are still to be clarified (Madsen and Bugge 2015). Cancer cells do express various types of matrix-degrading enzymes (Hanahan and Weinberg 2011; Weinberg 2006), but there are also other types of stromal cells that can express them, as well (Madsen and Bugge 2015).

One of the most abundant stromal cells is represented by the macrophage population, which can form up to 50% of tumour mass (Kelly et al. 1988; Vinogradov et al. 2014). Macrophages have been investigated in the past for their ability to degrade the various components of the ECM via the matrix metalloproteases (MMPs) that they can express (Werb et al. 1980; Vérollet et al. 2011; Madsen et al. 2017), as well as for their plasticity and anti-tumour/pro-tumour roles. In particular, macrophages are a very heterogeneous population, with the two extreme phenotypes represented by the classically activated anti-tumour M1 cells and the alternatively activated pro-tumour M2 cells (Mantovani et al. 2017; Sica et al. 2008). The tumour environment (i.e. the cytokines and chemokines in the environment) induces a transition from an initial M1-like macrophage phenotype to an M2-like macrophage phenotype, such that the advanced (detectable) tumours contain mostly cells with an M2-like phenotype. Moreover, a recent experimental study on the macrophages’ phenotype in response to ECM bioscaffolds suggested that such macrophages have an M2-like phenotype (Huleihel et al. 2017). In addition, an earlier study (Madsen et al. 2013) showed that the M2-like macrophages are responsible for the degradation of collagen, which is an important fibrous component of the ECM. Therefore, given the crucial role played by the ECM fibres such as collagen not only within the overall ECM architecture but also within individual and collective cell migration (Wolf and Friedl 2011; Wolf et al. 2013), understanding the role of macrophages and their interactive migratory dynamics with both the cancer cell population and the underlying ECM fibres distribution during ECM degradation and remodelling will bring important knowledge about the wider cancer invasion process. However, since in vitro and in vivo studies on macrophages–ECM–cancer interactions are still in their infancy, mathematical and computational approaches can help by generating new hypotheses about these interactions.

Over the last decades, various mathematical models have been used to investigate cell migration in cancer invasion and related processes, see Anderson et al. (2000, 2009), Anderson (2005), Chaplain and Lolas (2005, 2006), Deakin and Chaplain (2013), Dallon et al. (1999), Deisboeck et al. (2011), Domschke et al. (2014), Knútsdóttir et al. (2014), Mahlbacher et al. (2018), McDougall et al. (2006), Shuttleworth and Trucu (2019), Szymańska et al. (2009), Trucu et al. (2013) and references therein. The majority of these models focus on the interactions between the cancer cells and the ECM during the invasion process, with some models considering also the role of macrophages during cancer invasion (Knútsdóttir et al. 2014; Mahlbacher et al. 2018; Owen et al. 2004; Owen and Sherratt 1997, 1998; Webb et al. 2007). The earlier models focused mainly on the anti-tumour role of macrophages (Owen and Sherratt 1997, 1998; Webb et al. 2007), while the later models focused on the pro-tumour role of M2 macrophages (Knútsdóttir et al. 2014) and the anti-tumour/pro-tumour roles of M1/M2 macrophages (Mahlbacher et al. 2018) Moreover, while initially the mathematical models focused mainly on processes taking place at one spatial/temporal scale (Szymańska et al. 2009; Anderson et al. 2000; Chaplain and Lolas 2005, 2006), later the models started to acknowledge the multiscale dynamics of cancer progression. Indeed, the focus of recent modelling has shifted towards capturing the interplay between various processes that occur at different temporal and spatial scales (Deisboeck et al. 2011; Trucu et al. 2013; Shuttleworth and Trucu 2019), but despite the progress made in this regard, these models did not address also the contribution of the macrophages to the invasion process.

In this study, we build upon the multiscale moving-boundary modelling framework first introduced in Trucu et al. (2013) and later expanded in Shuttleworth and Trucu (2019) to account for the fibre and non-fibre components of the ECM. To that end, we consider the effects of both random and directional movement of M2-like macrophages not only on the remodelling of the ECM but also on the collective dynamics of the cancer cells, ultimately exploring their contribution on the overall tumour progression. The model introduced in Shuttleworth and Trucu (2019) and Trucu et al. (2013) is extended here as follows.

First, at macro-scale, we expand its macro-dynamics to capture also the contribution that the M2-like macrophages bring to the overall coupled dynamics. This will be carried out both by deriving another equation describing the spatio-temporal evolution of the density of the M2-like macrophages and by amending the cell dynamics to account for the interactions (including the formation of cell-adhesion bonds) with the macrophages. This will not only highlight the impact that the directionality of the macrophages dynamics has over the cancer cell invasion at macro-scale, but also explore its additional contribution towards the rearrangements of the ECM fibres at micro-scale (Shuttleworth and Trucu 2019). Indeed, the macrophages spatial dynamics and their interactions with the ECM fibres not only will affect their spatial bias (that naturally emerges to withstand incoming cell fluxes, as derived in Shuttleworth and Trucu (2019)) with direct impact on ECM remodelling, but in addition will also impact the growth and motility of tumour cells. Furthermore, the modelling approach that we propose here will also explore the dependence of both M2 macrophages and cancer on the stiffness of the ECM.

Second, at micro-scale, we will advance further the modelling proposed in Shuttleworth and Trucu (2019), by accounting both the contribution of the macrophages to the emergence of the leading edge cell-scale proteolytic dynamics occurring along the invasive edge of the tumour and their impact on the micro-scale spatial rearrangement of the ECM fibres microconstituents.

Finally, we use this new modelling approach that we propose in this paper to shed light on the importance of the directionality of macrophages dynamics within the multiscale nature cancer invasion in a fibrous tissue environment, ultimately bringing new understanding of this complex process.

The structure of the paper is as follows. We describe the new mathematical model in Sects. 2.1 (macro-scale dynamics) and 2.2 (micro-scale dynamics). In Sect. 3.1, we summarise the numerical scheme used to discretise this multiscale moving-boundary model. In the remainder of Sect. 3, we present a variety of simulations of cancer invasion within fibrous tissue environment, focusing on several biologically relevant settings for directional macrophages motility. Finally, we conclude with a summary and discussion of the results in Sect. 4.

## Multiscale Modelling of Tumour and Dynamics within Fibrous ECM

Building on the multiscale moving-boundary modelling approaches proposed for cancer invasion in Shuttleworth and Trucu (2019), Trucu et al. (2013), in this work we extend and advance this modelling platform by exploring further the macro- and micro-scale dynamics of the tumour invasion process. Specifically, in contrast to the situation addressed in Shuttleworth and Trucu (2019) and Trucu et al. (2013), we expand now the biological context by exploring the multiscale process of cancer cells invasion in the presence of tumour-associated macrophages with M2-like phenotype, shortly addressed here as M2 TAMs. This will extend the modelling presented in Shuttleworth and Trucu (2019) by incorporating here the dynamics of M2 TAMs population and the impact of its directional motility on overall tumour progression.

### Macro-Scale Dynamics

**Fig. 1:**
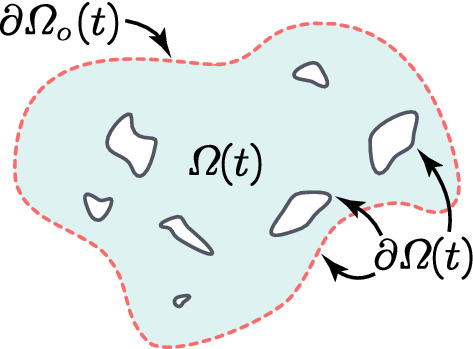
Illustration of the outer boundary $\partial \varOmega _{o}(t)$ that is highlighted with the *dashed red* line (Color figure online)

As this work builds on the multiscale modelling developed in Shuttleworth and Trucu (2019), Trucu et al. (2013), we start this section by revisiting some critical features of the framework. Hence, at macro-scale, we explore the cancer invasion process occurring within a maximal tissue cube $Y \in {\mathbb {R}}^{d}$ for $d = 2,3$, where the expanding tumour region denoted by $\varOmega (t)$ progresses over the time interval [0, *T*] (i.e. $\varOmega (t)\subset Y$, $\forall \,\, t\in [0,T]$). We adopt the same simplified context as in Trucu et al. (2013), Peng et al. (2017) and Shuttleworth and Trucu (2019, 2020a, 2020b) where aside from the tumour cells population *c*(*x*, *t*), the rest of the tumour micro-environment and surrounding tissue is represented here simply by a generic ECM. To that end, while we acknowledge that, besides the tumour cells, some of the tumour micro-environment components are not ECM constituents and are rather only supported by the usual ECM, in this framework we still regard all those constituents (such as VEGF, FGF, TGF-beta and ions such as $\hbox {Ca}^{2+}$) as being part of and represented by this extended concept of ECM. Furthermore, due to the biologically established importance played within cell migration by the major ECM fibres, namely collagen and fibronectin, as considered also in Shuttleworth and Trucu (2019, 2020a, 2020b), we regard this ECM as two-phase matter, consisting of an ECM fibre phase and an ECM non-fibre phase.Specifically, on one hand the ECM fibres phase accounts exclusively for all major fibres components such as collagen and fibronectin (notably characterised by their insolubility properties (Hynes and Naba 2012)), and its amount distributed at (*x*, *t*) is denoted here by *F*(*x*, *t*). On the other hand, besides the major fibres components, the ECM contains also an entire host of other soluble constituents, such as calcium ions Ca$^{2+}$ (Bhagavathula et al. 2007; Hofer et al. 2000), as well as other small proteins and soluble peptides that beyond a certain concentration threshold lead to the formation of insoluble amyloid fibrils (Rambaran and Serpell 2008), which notably have been found to support support cell adhesion (Ghosh et al. 2017; Gras 2009; Gras et al. 2008; Jacob et al. 2016). Thus, here all these ECM constituents that are not major fibres (i.e. these are neither collagen nor fibronectin) are bundled into a second ECM phase, and to immediately distinguish these from the ECM fibres, we simply refer to this phase as the *non-fibre ECM phase*. The spatio-temporal distribution of the non-fibre ECM phase at (*x*, *t*) is denoted by *l*(*x*, *t*). Furthermore, we also consider the presence of a population of M2-like TAMs, denoted here by *M*(*x*, *t*), which infiltrate the tumour as an immune response though the *outer boundary* that we denote by $\partial \varOmega _{o}(t)\subset \partial \varOmega (t)$, which is mathematically defined in “Appendix B” and is illustrated schematically in Fig. 1. Finally, for a compact notation, we denote by $\mathbf{u }$ the global four-dimensional tumour vector given by

1 $$\begin{aligned} \mathbf{u }(x,t) := (c(x, t), F(x, t), l(x, t), M(x, t))^{T}, \end{aligned}$$

and $\rho (\mathbf{u })$ represents the total space occupied at position *x*, i.e.,

2 $$\begin{aligned} \rho (\mathbf{u }) = c(x, t) + F(x, t) + l(x, t) + M(x, t), \end{aligned}$$

for all $t\in [0,T]$ and all $x\in \varOmega (t)$.

#### Tumour Cells Population Dynamics

Recent biological evidence shows that M2 TAMs macrophages enhance the cancer cell proliferation process (Goswami et al. 2017). Hence, assuming logistic-type growth for the cancer cell population [see Laird (1964, 1965), Tjorve and Tjorve (2017)], this enhancement brought in by the macrophages leads to an augmented proliferation for the cancer cells, which can be captured mathematically as:

3 $$\begin{aligned} P_{c}(\mathbf{u }):= \mu _{c} c [1+\mu _{cM}M][1-\rho (\mathbf{u })]^{+}. \end{aligned}$$

**Fig. 2:**
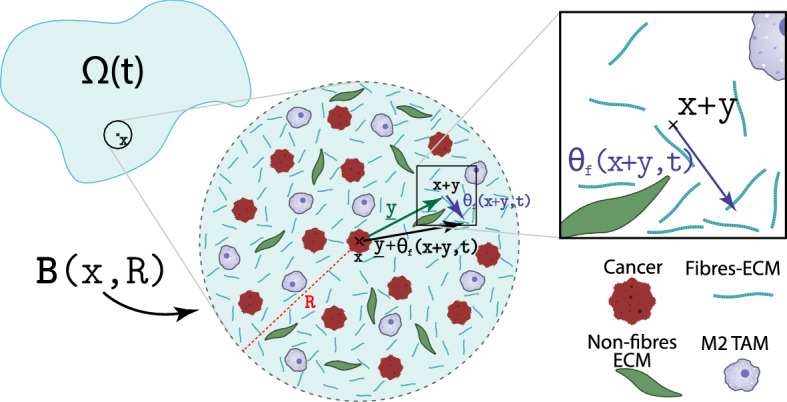
Schematics of the cell–fibre adhesion that is biased by the orientation of the fibres

Further, in the presence of proliferation (3), the tumour population *c*(*x*, *t*) exercises not only random movement (captured here through diffusion), but their spatial transport of the cell population is further amended and biased by a directional movement induced by the cell-adhesion processes (Huda et al. 2018; Petrie et al. 2009; Weiger et al. 2013; Wu et al. 2014). In this context, the spatio-temporal dynamics of the cancer cell population can therefore be expressed mathematically as:

4 $$\begin{aligned} \frac{\partial c}{\partial t} = \nabla \cdot \Big [ D_{c}\nabla c - c{{\mathcal {A}}_{c}}(x, t, \mathbf{u }, \theta _{f}) \Big ] +P_{c}(\mathbf{u }){,} \end{aligned}$$

where $D_{c}>0$ is a constant diffusion coefficient, and ${{\mathcal {A}}_{c}}(x, t, \mathbf{u }, \theta _{f})$ describes the cell-adhesion processes that bias the cancer cell population movement in accordance with the spatial heterogeneous distribution of the surrounding cancer cells, macrophages, and ECM components including the oriented ECM fibres. Specifically, in addition to the situation considered in Shuttleworth and Trucu (2019, 2020a, 2020b) (exploring the adhesive interactions of the cells distributed at $x\in \varOmega (t)$ with the other cancer cells as well as with the distribution of non-fibres ECM phase (Ghosh et al. 2017; Gras 2009; Gras et al. 2008; Jacob et al. 2016) and the oriented ECM fibres phase (Wolf et al. 2009; Wolf and Friedl 2011) within a sensing region ${\mathbf {B}}(x,R)$ of radius $R>0$), here the flux term ${\mathcal {A}}(x, t, \mathbf{u }, \theta _{f})$ explores the key biological evidence underlining the contribution of the macrophages to the directional movement of the tumour cells. Indeed, this not only explores the fact that cancer cells bind themselves to TAMs (Chen et al. 2011), but also accounts for the experimental evidence detailed in Condeelis and Pollard (2006), Green et al. (2009), Wei et al. (2019), Yamaguchi et al. (2006) that underscores the existence of a cross talk between tumour cells and macrophages which is mediated through various chemokines. Further, while we do not model here explicitly the involved chemokine activities, mathematically we account for this cross talk through the following non-local flux term:

5 $$\begin{aligned} \begin{aligned} {\mathcal {A}}_{c}(x, t, \mathbf{u }, \theta _{f})\! := \frac{1}{R}\!\! \int \limits _{{\mathbf {B}}(0,R)}\!\!\! \!\!\! {\mathcal {K}}(y)&\Big [ n(y) \big ( {\mathbf {S}}_\mathrm{cc} c(x\!+\!y, t)\\&\quad + {\mathbf {S}}_\mathrm{cM} M(x\!+\!y,t)+ {\mathbf {S}}_\mathrm{cl} l(x\!+\!y, t)\big ) \\&\quad + {\widehat{n}}(y, \theta _{f}(x\!+\!y,t)\!) {\mathbf {S}}_\mathrm{cF} F(x\!+\!y, t)\! \Big ]\! \big [ 1-\rho (\mathbf{u }) \big ]^{+}, \end{aligned} \end{aligned}$$

with the involved terms being detailed as follows. First, existing biological evidence (Gu et al. 2014; Hofer et al. 2000) (revealing the positive correlation between the availability of the extracellular Ca$^{+2}$ ions within the ECM and the strength of the adhesion bonds that the cancer cells are able to establish between themselves) enables us to assume that the cell–cell adhesion strength ${\mathbf {S}}_\mathrm{cc}$ depends on the non-fibre ECM density. Hence, proceeding here as in  Shuttleworth and Trucu (2019, 2020a, 2020b), we take ${\mathbf {S}}_\mathrm{cc}$ to be of the form

$$\begin{aligned} {\mathbf {S}}_\mathrm{cc}(x,t) := {\mathbf {S}}_{\min } + ({\mathbf {S}}_{\max }-{\mathbf {S}}_{\min }) \exp \bigg [ 1-\dfrac{1}{1-(1-l(x, t))^{2}} \bigg ] , \end{aligned}$$

which smoothly explores a full range of cell–cell cancer self-adhesion strengths, from its maximum level ${\mathbf {S}}_{\max }>0$ that corresponds to the Ca$^{+2}$-saturation level to its minimum values ${\mathbf {S}}_{\min }>0$ that corresponds to the minimum level of Ca$^{+2}$. Further, as on the sensing region the cell–cell cancer self-adhesion is complemented by an *adhesion* relationship between the cancer cells and macrophages, ${\mathbf {S}}_\mathrm{cM} > 0$ represents the combined strength of the cancer cell–macrophages adhesion. Finally, the cell–matrix adhesion manifests itself in this context through both adhesion between the cell and the ECM fibres (Wolf et al. 2009; Wolf and Friedl 2011) and adhesion between the cells and non-ECM fibre phase (which includes, for instance, densities of amyloid fibrils that have been proved experimentally to support cell adhesion (Ghosh et al. 2017; Gras 2009; Gras et al. 2008; Jacob et al. 2016)). The adhesion strength between cancer cells and non-fibre ECM and the adhesion strength between cancer cells and fibre ECM, denoted here by ${\mathbf {S}}_\mathrm{cl}$ and ${\mathbf {S}}_\mathrm{cF}$, respectively, are considered to be positive constants. Furthermore, $n(\cdot )$ represents here the unit radial vector given by

$$\begin{aligned} n(y) := {\left\{ \begin{array}{ll} \dfrac{y}{\parallel y \parallel } &{} \text {if } y \in {\mathbf {B}}(0, R) \setminus \{0\}, \\ 0 &{} \text {if } y = 0, \end{array}\right. } \end{aligned}$$

and ${\widehat{n}}(\cdot , \cdot )$ is the unit vector that is biased by the fibre orientations, i.e.

6 $$\begin{aligned} {\widehat{n}}(y, \theta _{f}(x+y,t)) := {\left\{ \begin{array}{ll} \dfrac{y+\theta _{f}(x+y,t)}{\parallel y+\theta _{f}(x+y,t) \parallel } &{} \text {if } y \in {\mathbf {B}}(0, R) \setminus \{0\}, \\ 0 &{} \text {if } y = 0, \end{array}\right. } \end{aligned}$$

where $\theta _{f}(x,t)$ is the orientation of the fibres at macro-scale that was derived and introduced for the first time in Shuttleworth and Trucu (2019), being derived by exploring the structural micro-scale mass distribution of their constituent micro-fibres and characterising the spatial bias of the ECM fibres distributed at the macro-scale location $x\in Y$, see for details Sect. 2.2. Figure 2 shows one of the biased vectors $y + \theta _{f}(x+y,t)$, with $y\in {\mathbf {B}}(0,R)$, that are involved in (6), illustrating the way in which the orientation of the ECM fibres bias the direction of vector *y*, crucially influencing the cell–fibre adhesion process.

**Fig. 3:**
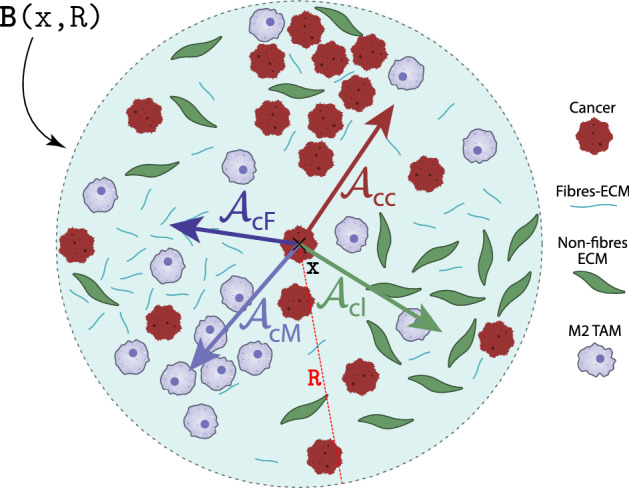
Schematics of adhesion process inside the sensing region ${\mathbf {B}}(x,R)$. In order to illustrate this process, the adhesion term ${\mathcal {A}}_{c}{(\cdot ,\cdot ,\cdot ,\cdot )}$ given in (5) is appropriately given as the sum of four main constituents, namely ${\mathcal {A}}_\mathrm{cc}$, ${\mathcal {A}}_\mathrm{cl}$, ${\mathcal {A}}_\mathrm{cF}$ and ${\mathcal {A}}_\mathrm{cM}$ that correspond to cell–cell, cell-non ECM fibres, cell-ECM fibres adhesions and cell-macrophage adhesion contributions, respectively. Here, we envisaged four regions inside the sensing region ${\mathbf {B}}(x,R)$ where one of the tumour components (i.e. either cancer cells, or ECM fibres or ECM non-fibres or M2 TAMs) is predominant and forms a local majority in terms of their spatial distribution versus the other three. In this context, the vector ${\mathcal {A}}_\mathrm{cl}$ is pointing from the centre of the sensing region towards the non-fibre group because the non-local bonds are the strongest towards that direction. We see a similar behaviour for ${\mathcal {A}}_\mathrm{cc}$, ${\mathcal {A}}_\mathrm{cF}$ and ${\mathcal {A}}_\mathrm{cM}$. However, the fibre adhesion is biased by the orientation of the fibres, and so the vector points towards a biased direction; see Fig. 2. On the other hand, since ${\mathbf {S}}_\mathrm{cc}$ depends on the density of non-fibre ECM, we observe that ${\mathcal {A}}_\mathrm{cc}$ is aided by the position of the region where we have the grouping of the non-fibre component. Finally, ${\mathcal {A}}_\mathrm{cM}$ points from the centre point *x* towards the groupings of M2 TAMs due to adhesion. Adding these elements together yields ${\mathcal {A}}_{c}{(x, t, \mathbf{u }, \theta _{f})}$ that is given by (5), and so we expect the mass of tumour cells distributed at position *x* to move towards this direction

To account in (5) also for the gradual weakening of the adhesion between cancer cells and macrophages as well as of the adhesive bonds between the cancer cells at $x\in \varOmega (t_{0})$ and the cells and ECM fibre and non-fibre phases as we move away from the location *x* within ${\mathbf {B}}(x,R)$, we use a radially symmetric ${\mathcal {K}}(\cdot )$ that is given here by

7 $$\begin{aligned} {\mathcal {K}}(y)=\psi \left( \frac{y}{R}\right) , \qquad \forall y\in {\mathbf {B}}(0,R), \end{aligned}$$

where $\psi (\cdot )$ is the standard mollifier defined in Appendix C. Finally in (5), $(1-\rho (\mathbf{u }))^{+} = \max (0, 1-\rho (\mathbf{u }))$ ensures that overcrowded tumour sites do not contribute to the migration of the cancer cells. To conclude, Fig. 3 illustrates the way the adhesion flux ${\mathcal {A}}_{c}(x, t, \mathbf{u }, \theta _{f})$ emerges, as this can be regarded as a sum of four different adhesion contributors, namely: ${\mathcal {A}}_\mathrm{cc}$ for cell–cell cancer self-adhesion, ${\mathcal {A}}_\mathrm{cM}$ for cell-macrophage adhesion, ${\mathcal {A}}_\mathrm{cl}$ for cell-non-fibre adhesion and ${\mathcal {A}}_\mathrm{cF}$ for cell–fibre adhesion, which are given by:

$$\begin{aligned} {\mathcal {A}}_\mathrm{cc}(x, t, \mathbf{u }, \theta _{f})&:=\frac{1}{R} \int \limits _{{\mathbf {B}}(0,R)} {\mathcal {K}}(y) n(y) \big ( {\mathbf {S}}_\mathrm{cc} c(x+y, t) \big ) \big [ 1-\rho (\mathbf{u }) \big ]^{+},\\ {\mathcal {A}}_\mathrm{cM}(x, t, \mathbf{u }, \theta _{f})&:=\frac{1}{R} \int \limits _{{\mathbf {B}}(0,R)} {\mathcal {K}}(y) n(y) \big ( {\mathbf {S}}_\mathrm{cM} M(x+y, t) \big ) \big [ 1-\rho (\mathbf{u }) \big ]^{+},\\ {\mathcal {A}}_\mathrm{cl}(x, t, \mathbf{u }, \theta _{f})&:=\frac{1}{R} \int \limits _{{\mathbf {B}}(0,R)} {\mathcal {K}}(y) n(y) \big ( {\mathbf {S}}_\mathrm{cl} l(x+y, t) \big ) \big [ 1-\rho (\mathbf{u }) \big ]^{+},\\ {\mathcal {A}}_\mathrm{cF}(x, t, \mathbf{u }, \theta _{f})&:=\frac{1}{R} \int \limits _{{\mathbf {B}}(0,R)} {\mathcal {K}}(y) {\widehat{n}}(y) \big ( {\mathbf {S}}_\mathrm{cF} F(x+y, t) \big ) \big [ 1-\rho (\mathbf{u }) \big ]^{+}.\\ \end{aligned}$$

#### Macro-Scale Dynamics of the Fibres and Non-fibre ECM Phases

Based on biological evidence that the components of non-fibre ECM phase (e.g. the amyloid fibrils) are degraded by several classes of matrix metalloproteinases (Stix et al. 2001; Liao and Van Nostrand 2010), we extend here the context considered in Shuttleworth and Trucu (2019, 2020a, 2020b), where this degradation was considered to be caused only by the MMPs secreted by the cancer cells. So now we consider not only the contribution of cancer cells but also that of the macrophages to the secretion of the various classes of matrix metalloproteinases (Aristorena et al. 2019; Huang et al. 2012). Hence, the degradation of the non-fibre ECM phase is caused indirectly (through the secretion of MMPs) not only by the tumour cells, at rate $\lambda _\mathrm{lc} > 0$, but also by the M2 TAMs, at rate $\lambda _\mathrm{lM} > 0$. Furthermore, while depending on the free space available, the remodelling of the ECM is also enhanced by the presence of the macrophages (Afik et al. 2016; Goswami et al. 2017; Springer and Fischbach 2016). Hence, the dynamics of the non-fibre component *l*(*x*, *t*) is described by

8 $$\begin{aligned} \frac{\partial l}{\partial t} = -l(\lambda _\mathrm{lc}c + \lambda _\mathrm{lM}M) + {(\alpha _{1}+\alpha _{2} M)}(1-\rho (\mathbf{u })), \end{aligned}$$

where $\alpha _{1}$ is the remodelling rate in the absence of *M*, and $\alpha _{2}$ represents the remodelling enhancement rate enabled by the presence of *M*.

Finally, the macroscopic ECM fibres *F*(*x*, *t*) are degraded both by the cancer cells and by the macrophages (with collagen-endocytosing TAMs being one of the main contributors towards the degradation of collagen in tumours, according to Madsen et al. (2017)). Thus, their dynamics can be mathematically formalised as

9 $$\begin{aligned} \frac{\partial F}{\partial t} = -F(\gamma _\mathrm{Fc}c + \gamma _\mathrm{FM}M), \end{aligned}$$

where $\gamma _\mathrm{Fc}$ and $\gamma _\mathrm{FM}$ are the ECM fibres degradation rates associated with cancer cells and macrophages, respectively.

#### M2 Macrophage

The last macro-scale tumour constituent that we consider in this work is the family of M2 TAMs macrophages *M*(*x*, *t*). To describe the macrophages dynamics, we note that the experimental study in Redente et al. (2007) showed that: (i) the number of peritumoral macrophages increased during oncogeny, (ii) the macrophages entering the circulation from the bone marrow (to reach the tumour site) already had a M2-phenotype, likely due to tumour-derived biochemical signals (Redente et al. 2007). Moreover, the experimental study in Green et al. (2009) showed that TAMs localise at the invasive area of the tumour, where they secrete cytokines and proteases that contribute to tumour invasion. Therefore, in this study we assume that the M2-like macrophages enter the tumour through blood vessels at the tumour boundary, at a constant influx density $M_{0}$. Denoting the tumour boundary by $\varOmega _{o}(t)$, we define the M2 influx term as follows:

10 $$\begin{aligned} M_{I}(x, t) := M_{0}(\chi _{_{\partial \varOmega _{o}(t)}}*\psi _{\rho } )(x). \end{aligned}$$

Here, $\chi _{_{\partial \varOmega _{o}(t)}}$ represents the characteristic function of the outer boundary $\partial \varOmega _{o}(t)$ (defined in Appendix B), and $\psi _{\rho }(\cdot )$ denotes the standard mollifier given by $\psi _{\rho }(x):=\frac{1}{\rho ^{d}}\psi (x)$ with a small cell-scale mollification range $\rho >0$.

Regarding macrophages proliferation, on the one hand biological evidence (Cassetta et al. 2019; Chitu et al. 2011; Jenkins et al. 2011) shows that cancer cells trigger this proliferative process through the production of survival and proliferation factors. On the other hand, the ECM stiffness was also shown to enhance macrophages proliferation (Adlerz et al. 2016). Furthermore, based on the biological evidence presented in Provenzano et al. (2009), where it was demonstrated experimentally that increased collagen matrix density increases matrix stiffness (a fact that was further confirmed in Mierke (2011), Wullkopf et al. (2018)), there exists a direct correlation between the ECM stiffness and the ECM fibre phase density. Hence, this direct correlation enables us to assume here not only that the ECM stiffness is directly proportional to the ECM fibre phase density, but in fact that the ECM stiffness is actually given directly by *F*(*x*, *t*) (i.e., the proportionality constant is considered here to be 1). Thus, the proliferation law of the macrophages can therefore be mathematically formulated as

11 $$\begin{aligned} P_{M}(\mathbf{u }):=\mu _{M} M c(1 + \mu _\mathrm{MF}F) (1 - \rho (\mathbf{u }))^{+}, \end{aligned}$$

where $\mu _{M} > 0$ is the baseline proliferation rate, $\mu _\mathrm{MF} > 0$ is the enhancement rate of the fibres and $(1 - \rho (\mathbf{u }))^{+}$ ensures that there is no overcrowding.

Similar to the cancer cells, macrophages exercise not only random movement but also directed migration. Hence, we account for the random movement part via diffusion with a coefficient $D_{M} > 0$ (which for the time being is considered constant), while their directed movement is captured through an *adhesion* term that is similar to the one that we used for the cancer cell population in (5). Specifically, we consider here a cell–cell M2 TAMs self-adhesion with constant strength ${\mathbf {S}}_\mathrm{MM} > 0$ and a macrophage–cancer cells adhesion with strength ${\mathbf {S}}_\mathrm{Mc} > 0$ (we have already mentioned in Sect. 2.1.1 that cancer cells can bind themselves to TAMs, in addition to attracting TAMs, as shown in Condeelis and Pollard (2006), Dutta et al. (2018), Xuan et al. (2014)). Finally, since we aim to explore the various factors that may affect macrophage movement, we also consider macrophage–fibre ECM adhesion with strength ${\mathbf {S}}_\mathrm{MF} > 0$. Hence, the macrophage adhesion term ${\mathcal {A}}_{M}(x,t,\mathbf{u },\cdot )$ is given by

12 $$\begin{aligned} \begin{aligned} {\mathcal {A}}_{M}(x, t, \mathbf{u }, \theta _{f})\! := \frac{1}{R}\!\! \int \limits _{{\mathbf {B}}(0,R)}\!\!\! \!\!\!{\mathcal {K}}(y)&\Big [ n(y) \big ( {\mathbf {S}}_\mathrm{MM} M(x\!+\!y, t)+ {\mathbf {S}}_\mathrm{Mc} c(x\!+\!y,t)\big ) \\&\quad + {\widehat{n}}(y, \theta _{f}(x\!+\!y,t)\!) {\mathbf {S}}_\mathrm{MF} F(x\!+\!y, t)\! \Big ]\! \big [ 1-\rho (\mathbf{u }) \big ]^{+}. \end{aligned} \end{aligned}$$

Here, *R* is the radius of the sensing region ${\mathbf {B}}(x,t)$, ${\mathcal {K}}(\cdot )$ is the diffusion kernel defined in (7) to model the weakening effect, $[ 1-\rho (\mathbf{u }) \big ]^{+}$ guarantees that overcrowded regions do not have any adhesion contribution towards the overall macrophages dynamics, and $\theta _{f}(x+y,t)$ is the orientation of the fibres on the macro-scale (see details in Sect. 2.2). Due to the similarities between the structure of the two adhesion fluxes, i.e., ${\mathcal {A}}_{M}(x, t, \mathbf{u }, \theta _{f})$ given in (5) and ${\mathcal {A}}_{c}(x, t, \mathbf{u }, \theta _{f})$ given in (12), we refer the reader to Figs. 2 and 3 for illustration of the macrophage adhesion term (12).

Therefore, the dynamics of the M2-like macrophages *M*(*x*, *t*) is given by

13 $$\begin{aligned} \frac{\partial M}{\partial t} = \nabla \cdot [D_{M} \nabla M - M{\mathcal {A}}_{M}(x, t, \mathbf{u }, \theta _{f})] - d_{M} M + M_{I} + P_{M}(\mathbf{u }), \end{aligned}$$

where $d_{M} > 0$ represents the natural macrophages death rate.

##### Remark 1

Since various experimental studies discuss the distribution of M2 TAMs macrophages inside tumour tissue and its prognostic value (Liu et al. 2017; Sumimoto et al. 2019), here we focus exclusively on the tumour–macrophage interactions on the tumour domain, rather than considering these mixed within the ECM further afield outside the tumour. This is another reason for which our modelling assumptions assume a macrophage influx through tumour boundary (see Eq. (10)).

#### Summary of the Macro-Dynamics

In summary, using (4), (8), (9) and (13), the coupled PDEs system that describes our macro-scale cancer invasion dynamics is given by

14a $$\begin{aligned} \frac{\partial c}{\partial t}&= \nabla \cdot \Big [ D_{c}\nabla c - c{\mathcal {A}}(x, t, \mathbf{u }, \theta _{f}) \Big ] +P_{c}(\mathbf{u }), \end{aligned}$$

14b $$\begin{aligned} \frac{\partial F}{\partial t}&= -F(\gamma _\mathrm{Fc}c + \gamma _\mathrm{FM}M), \end{aligned}$$

14c $$\begin{aligned} \frac{\partial l}{\partial t}&= -l(\lambda _\mathrm{lc}c + \lambda _\mathrm{lM}M) + (\alpha _{1}+\alpha _{2} M)(1-\rho (\mathbf{u })), \end{aligned}$$

14d $$\begin{aligned} \frac{\partial M}{\partial t}&= \nabla \cdot [D_{M} \nabla M - M{\mathcal {A}}_{M}(x, t, \mathbf{u }, \theta _{f})] - d_{M} M + M_{I} + P_{M}(\mathbf{u }), \end{aligned}$$

with zero-flux boundary conditions. Finally, in Table 1, we summarise the adhesion effects between the different constituents that were considered throughout this section.

**Table 1:** Summary of all adhesion processes that we consider in our macro-scale dynamics (14)

	Cancer cells	M2 TAMs	Non-fibre ECM	Fibre ECM
Cancer cells	${\mathbf {S}}_\mathrm{cc}$	${\mathbf {S}}_\mathrm{cM}$	${\mathbf {S}}_\mathrm{cl}$	${\mathbf {S}}_\mathrm{cF}$
M2 TAMs	${\mathbf {S}}_\mathrm{Mc}$	${\mathbf {S}}_\mathrm{MM}$	0	${\mathbf {S}}_\mathrm{MF}$
Non-fibre ECM	0	0	0	0
Fibre ECM	0	0	0	0

### Processes on the Micro-Scales and Their Links to Macro-Scale

The macro-scale cancer invasion process is accompanied by several closely linked micro-dynamic processes (Weinberg 2006). Particularly important are those micro-processes concerning the cell-scale spatial redistribution of the ECM fibres micro-constituents caused by the interaction with the cancer cell population, and the cell-scale proteolytic processes occurring at the leading edge of the tumour. While different in nature, both of these micro-processes are intrinsically linked to the same macro-process, and in the following will explore the details of these micro-dynamics together with the associated double feedback links that connects them to the invasive tumour macro-dynamics.

#### Fibres: Their Micro-Scale Structure and Macro-Scale Representation

As discussed in detail in Shuttleworth and Trucu (2019), it is important to observe that the macroscopic ECM fibres are not only represented through their amount *F*(*x*, *t*) distributed at $(x,t)\in \varOmega (t)\times [0,T]$, but also through their naturally emerging spatial bias to withstand incoming cell fluxes and forces. This spatial bias is induced by their micro-scale distribution of micro-fibres *f*(*z*, *t*) on a cell-scale micro-domain $\delta Y(x):=x+\delta Y$ of appropriate micro-scale size $\delta >0$. Indeed, as derived and formalised in Shuttleworth and Trucu (2019), these two important characteristics of the ECM fibres (i.e. the amount of distributed ECM fibres and their associated spatial bias at (*x*, *t*)) can be captured simultaneously through a vector field representation of the ECM fibres, $\theta _{f}(x,t)$, that is mathematically expressed as

15 $$\begin{aligned} \theta _{f}(x,t):= \dfrac{1}{\lambda (\delta Y(x))} \int \limits _{\delta Y(x)} f(z, t) \; dz \cdot \dfrac{\theta _{f, \delta Y(x)} (x, t)}{\parallel \theta _{f, \delta Y(x)} (x, t) \parallel _{2}}. \end{aligned}$$

Here, $\lambda$ is the Lebesgue measure in ${\mathbb {R}}^{d}$ and $\theta _{f, \delta Y(x)} (x, t)$ is the revolving barycentral orientation with respect to the measure $f(z,t) \lambda (\cdot )$ that is induced and uniquely defined by the mass distribution of micro-fibres on the cell-scale micro-domain $\delta Y(x)$. This is given by the Bochner mean value (Yosida 1980) of the *barycentral vector-valued function* $\delta Y(x)\ni z\mapsto z-x \in {\mathbb {R}}^{d}$, namely

16 $$\begin{aligned} \theta _{f, \delta Y(x)} (x, t) := \dfrac{\int \limits _{\delta Y(x)} f(z, t)(z-x) \; dz}{\int \limits _{\delta Y(x)} f(z, t) \; dz}. \end{aligned}$$

Finally, the magnitude of the ECM fibres is given by the Euclidean norm of $\theta _{f}(x,t)$, i.e.

$$\begin{aligned} F(x,t):=\parallel \theta _{f}(x,t) \parallel _{2}, \end{aligned}$$

and represents the mean value of micro-fibres distributed on $\delta Y(x)$ at time $t\in [0,T]$.

#### Fibre Rearrangement

Under the incidence of the macro-scale spatial flux generated by the tumour macro-dynamics, the rearrangement of the ECM fibres takes place on each micro-domain $\delta Y(x)$ through the spatial re-distribution of its micro-fibres constituents. This process is instigated not only by the emerging macro-scale cancer cell flux ${\mathcal {F}}_{c}$ (as considered in Shuttleworth and Trucu (2019)), but also by the spatial flux of migrating M2 TAMs macrophages ${\mathcal {F}}_{M}$, these being defined by:

17 $$\begin{aligned} \begin{aligned} {\mathcal {F}}_{c}(x, t)&{:=}\, D_{c}\nabla c(x, t) - c(x, t) {\mathcal {A}}_{c}(x,t,\mathbf{u }, \theta _{f}), \\ {\mathcal {F}}_{M}(x, t)&{:=}\, D_{M}\nabla M(x, t) - M(x, t) {\mathcal {A}}_{M}(x,t,\mathbf{u }, \theta _{f}). \end{aligned} \end{aligned}$$

Therefore, at any spatio-temporal node (*x*, *t*), the combined spatial flux ${\mathcal {F}}_{c}(x, t)+{\mathcal {F}}_{M}(x, t)$ that acts uniformly upon the fibres distributed on the micro-domain $\delta Y(x)$ results in the emergence of a *micro-fibres rearrangement vector* $r(\delta Y(x), t)$ given by

18 $$\begin{aligned} r(\delta Y(x), t) := \omega _{c}(x, t) {\mathcal {F}}_{c}(x, t) + \omega _{M}(x, t) {\mathcal {F}}_{M}(x, t) + \omega _{F}(x, t) \theta _{f}(x, t). \end{aligned}$$

This rearrangement vector acts upon the mass distribution of the micro-fibres *f*(*z*, *t*) on $\delta Y(x)$, causing these to be spatially redistributed both on $\delta Y(x)$ and on the neighbouring micro-domains. The weights considered in (18), $\omega _{c}(x, t)$, $\omega _{M}(x, t)$ and $\omega _{F}(x, t)$, are appropriately given by the associated mass fractions of cancer cells, macrophages, and ECM fibres distributed at at (*x*, *t*), namely:

$$\begin{aligned} \begin{array}{c} \omega _{c}(x, t) := \frac{c(x, t)}{{c(x, t) + F(x, t) + M(x, t)}}, \quad \omega _{M}(x, t) := \frac{M(x, t)}{{c(x, t) + F(x, t) + M(x, t)}}, \\ \omega _{F}(x, t) := \frac{F(x, t)}{{c(x, t) + F(x, t) + M(x, t)}}. \end{array} \end{aligned}$$

Finally, for any micro-scale position $z\in \delta Y(x)$, we calculate the new position $z^{*}$ by using the *relocation vector* $\nu _{\delta Y(z)}(z,t)$:

$$\begin{aligned} z^{*} := z + \nu _{\delta Y(z)}(z,t). \end{aligned}$$

**Fig. 4:**
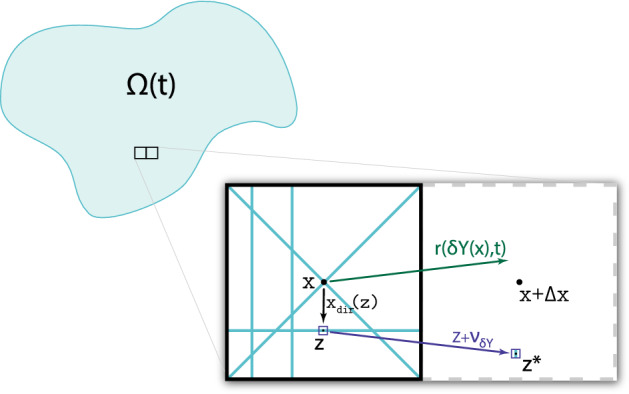
Sketch of the micro-fibre reallocation to the neighbouring fibres micro-domain and the vectors required for the process

In Fig. 4, we consider a typical example of these vectors to illustrate the process. For further details, we refer the reader to Appendix E or Shuttleworth and Trucu (2019).

#### Boundary Micro-Scale

The second type of micro-dynamics that we consider here is that exercised by the proteolytic molecular processes occurring along the invasive edge of the tumour. Biological evidence suggest that besides cancer cells, TAMs also produce matrix-degrading enzymes (MDEs), such as matrix metalloproteinases (MMP) of both type 2 (MMP-2) and type 9 (MMP-9) molecules (Goswami et al. 2017), which are essential for tumour progression. Secreted by the cancer cells and macrophages within the outer proliferating rim of the tumour, these molecules exercise a cross-interface transport within a cell-scale neighbourhood of the tumour interface, leading to degradation of the ECM in the peritumoural region, ultimately resulting in changes of the tumour boundary morphology and subsequent further tumour progression.

**Fig. 5:**
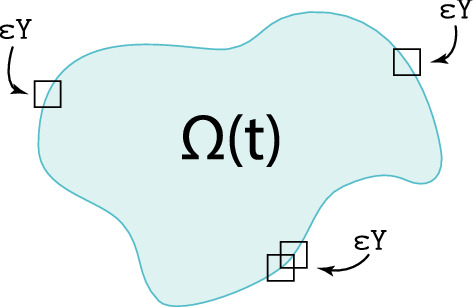
Schematics of the overlapping $\epsilon$-cubes that covers the boundary $\partial \varOmega (t_{0})$

To explore this emerging proteolytic micro-dynamics, we adopt the approach initially developed and introduced in Trucu et al. (2013). Specifically, we denote by $m(\cdot , \cdot )$ the spatio-temporal distribution of MDEs that are transported within a cell-scale neighbourhood of $\partial \varOmega (t)$. This neighbourhood is represented by the union of an appropriately constructed covering bundle of overlapping micro-domains $\{\epsilon Y\}_{\epsilon Y\in {\mathcal {P}}(t)}$ illustrated in Fig. 5, which enable us to decompose the overall boundary dynamics into a union of proteolytic micro-dynamics taking place on each $\epsilon Y$. Thus, for any macroscopic time $t_{0} \in [0,T]$ on any boundary micro-domain $\epsilon Y$, at any spatio-temporal location $(y,\tau )\in (\epsilon Y\cap \varOmega (t_{0}))\times [t_{0},t_{0}+\Delta t]$, a source $h(\cdot , \cdot )$ of MDEs arises as a collective contribution of both the cancer cells and the macrophages distributed within the tumour’s outer proliferating rim within a distance $\gamma >0$ from *y*. This source can be mathematically formulated as:

19 $$\begin{aligned} \begin{aligned} h(y, \tau )&= \dfrac{\int \limits _{{\mathbf {B}}(y, \gamma ) \cap \varOmega (t_{0})} \alpha _{c} c(x, t_{0} + \tau ) + \alpha _{M} M(x, t_{0} + \tau ) dx}{\lambda ({\mathbf {B}}(y, \gamma ) \cap \varOmega (t_{0}))}, \qquad y \in \epsilon Y \cap \varOmega (t_{0}), \\ h(y, \tau )&= 0, \qquad y \notin \epsilon Y \setminus (\varOmega (t_{0}) + \{ z \in Y \; | \; \Vert z \Vert _{2} < \rho \}), \end{aligned} \end{aligned}$$

where $\alpha _{c}$ and $\alpha _{M}$ are the MDE secreting rate of the cancer and the M2 TAMs, respectively. Further, ${\mathbf {B}}(y, \tau ) := \{ z \in Y \; | \; \Vert y - z \Vert _{\infty } \le \gamma \}$ denotes the $\parallel \cdot \parallel _{\infty }$ ball with appropriately chosen radius $\gamma >0$ where the source of MDEs is accumulated, and $0<\rho <\gamma$ is a small mollification range that smooths out the source $h(\cdot , \cdot )$ along the tumour interface.

Finally, the source $h(\cdot , \cdot )$ defined in (19) allows us to formulate the MDE micro-dynamics

20 $$\begin{aligned} \dfrac{\partial m}{\partial t} = D_{m}\Delta m + h(y, \tau ), \end{aligned}$$

which ultimately enables us to determine the movement of this interface. For further details, see “Appendix F” or Trucu et al. (2013).

### Summary of the Links Between the Scales

**Fig. 6:**
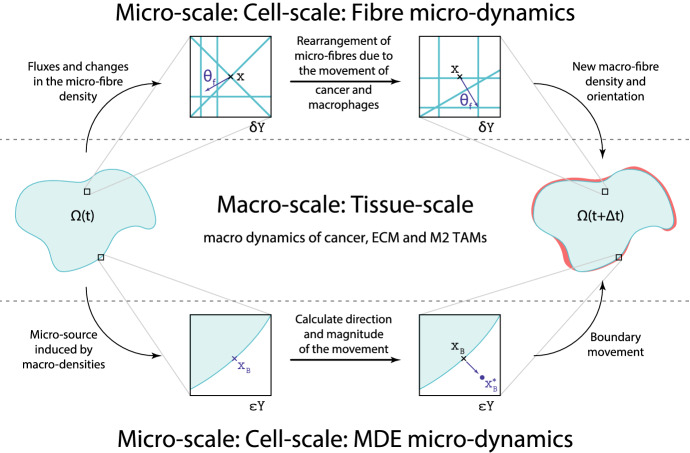
Links between the scales, and how the scales affect each other, in particular, the link between boundary micro- and macro-scales as well as fibre micro- and macro-scales

In summary, both micro-scales have their unique link to the macro-scale and vice versa. First, we recall that the spatial fluxes defined in (17) result in a vector field induced by the movement of both the cancer cells and M2 TAMs. This spatial flux interacts in a weighted manner with the oriented fibres (represented here again as a vector field), ultimately enabling the rearrangement of distribution of the micro-scale constituents of the fibres (referred to as micro-fibres), resulting in a changed spatial orientation of the ECM fibres. This establishes a *fibres top-down* link and it is illustrated in Fig. 6. On the other hand, the freshly rearranged micro-fibre density translates into a change in density as well as the orientation of the ECM fibres observed at the macro-scale, as detailed in Sect. 2.2.1, which in turn has a major impact upon the macro-scale dynamics (14). This establishes now a *fibres bottom-up* link that connects the fibre micro-scale to the macro-dynamics.

Shifting our focus to the relation between the boundary micro-dynamics and the macro-dynamics, the source of the MDEs (19) on the micro-scale is induced by the macro-scale population of cancer cells and macrophages. Under the presence of this source, the micro-dynamics (20) takes place within a cell-scale neighbourhood enabled by a covering bundle of overlapping boundary micro-domains $\{\epsilon Y\}_{\epsilon Y\in {\mathcal {P}}(t)}$, and their solution on each of $\epsilon Y$ ultimately enables us to determine a direction and magnitude for the movement of the tumour boundary captured by $\epsilon Y$. The local expansion of the tumour domain in the direction and by the magnitude determined from the proteolytic boundary micro-dynamics is finally exercised provided that a significant but not complete level of ECM degradation within the peritumoural tissue neighbourhood is realised. A quantification of this significant but not complete level of peritumoural degradation (which translates in the most favourable tissue conditions for invasion) is explored through a tissue parameter $\beta \in (0,1)$ that has been introduced and formalised in Trucu et al. (2013) and is briefly described in “Appendix F”, but for full details, we refer the reader to this initial reference. Thus, this connection between the boundary micro-dynamics and macro-dynamics is again connected through a *top-down*–*bottom-up* double feedback loop that is illustrated in the bottom half of Fig. 6.

## Numerical Approach and Computational Simulations

### Brief Overview of the Numerical Approach

In this section, we briefly discuss the numerical methods used to solve the macro-scale dynamics (14) and give an overview of the numerical approaches used for both fibres and MDE micro-scale dynamics (detailed in Sects. 2.2.2 and  2.2.3, respectively).

Let us start the discussion by focusing on the macro-scale dynamics (14), where we introduced the non-constant diffusion coefficients. Here, we use the method of lines (MOL) approach to first discretise the system in space, and then, for the time-marching we use the non-local predictor–corrector scheme introduced in Shuttleworth and Trucu (2019) (a new two-step time-splitting method involving the Euler method as the predictor and a non-local Trapezoidal-type rule as the corrector). In this context, the spatial discretisation of (14) is carried out on a uniform grid, where we accurately approximate the two distinct spatial operators, namely the diffusion and adhesion operators. While for the former one, we use a convolution-based second-order central difference scheme (as detailed in Appendix G.1.1), for the adhesion operators, we follow Domschke et al. (2014), Gerisch and Chaplain (2006, 2008) to construct a second-order finite-difference flux limiter scheme, (as described in “Appendix G.1.2”). Furthermore, to efficiently and accurately approximate the adhesion integrals introduced in (5) and (12), we use a convolution-driven approach. Here, we partition the sensing region ${\mathbf {B}}(0,R)$ into annulus sectors, which allows us to approximate the integrals in (5) and (12), by using the integral of the step functions associated with each annulus sector. For completeness, we present the details of this scheme in Appendix G.

Focusing now on the MDE micro-scale (detailed in Sect. 2.2.3), we first need to approximate the MDE source (19). For efficiency, we again use convolution to carry out the approximation of this integral, which we then interpolate on each MDE micro-domain $\epsilon Y$, enabling us to use it in the MDE micro-dynamics (20). The numerical scheme of this micro-dynamics again follows the MOL. So, we first discretise (20) in space using the second-order central difference scheme, and then the resulting ODEs are solved by the backward Euler time integration technique. This allows us to solve the MDE micro-dynamics on each $\epsilon Y$ which ultimately leads to a new expanded invasive tumour domain $\varOmega (t+\Delta t)$ for the macro-dynamics (14). For further details on the numerical technique used for the MDE micro-scale dynamics, we refer the reader to Appendix G.3.

Finally, the redistribution of the micro-fibres constituents of the ECM (detailed in Sect. 2.2.2) is performed on each fibre micro-domain $\delta Y (x)$. Hence, by using the spatial fluxes (generated by the cancer cells and macrophages), on each $\delta Y (x)$ we construct the emerging rearrangement vector which then induces a reallocation vector for each micro-node in $\delta Y (x)$. Then, using these reallocation vectors, we calculate the new positions of each micro-fibres, eventually leading to the new rearranged fibres ECM. For completeness, we present the details of this process in “Appendix E”.

### Initial Conditions

For the numerical simulations, we consider a spatial domain $Y=[0,4] \times [0,4]$. We start with the following initial conditions

21 $$\begin{aligned} \begin{array}{lll} c(x, 0) &{} =&{} 0.2 \cdot \chi _{_{{\mathbf {B}}((2,2), 0.25)}}(x), \\ l(x, 0) &{} = &{}\min \bigg ( \dfrac{1}{2} + \dfrac{1}{4} \sin (7 \pi x_{1}x_{2})^{3} \cdot \sin \big (7 \pi \dfrac{x_{2}}{x_{1}} \big ), 1 - c(x, 0) \bigg ), \\ M(x, 0) &{} = &{} \dfrac{1}{2} \cdot c(x,0). \end{array} \end{aligned}$$

**Fig. 7:**
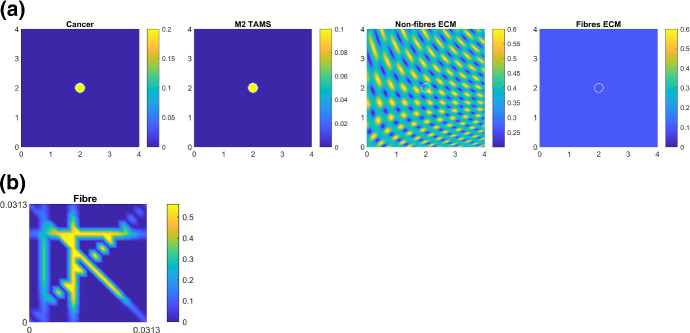
**a** Initial conditions for the macro-scale densities, i.e. for cancer, non-fibre ECM and fibres ECM defined in (21). **b** The initial condition of one micro-fibre domain which is repeated for every point on the macro-scale

These macro-scale initial conditions can be seen in Fig. 7a. Here, the white curves indicate the boundary of the tumour. In Fig. 7b, we show the initial condition for one micro-scale fibre domain $\delta Y (x)$, which is repeated for all macro-scale points. Note that the pattern of the fibres on the micro-scale is not visible on the macro-scale because in order to get the density of the fibres at any macro-point *x*, we integrate the corresponding fibre-micro domain $\delta Y (x)$ seen in Fig. 7b. We note here that the ratio between the fibres and non-fibres components of ECM is assumed to be $20\%$:$80\%$.

In this section, we present all of our simulations at time $50 \Delta t$, using the parameter values from the set ${\mathcal {S}}$ described in Appendix A which we regard as baseline, and any departure from these values will be stated accordingly.

We note here that any small differences in the model outcomes will be exclusively the result of changes in the parameter values, and not of any model stochasticity, as all equations and initial conditions are deterministic.

### Simulation Results

In this section, we investigate numerically the dynamics of the macro-scale model (14), where both cancer and macrophage diffusions are constants.

*Baseline Dynamics*

**Fig. 8:**
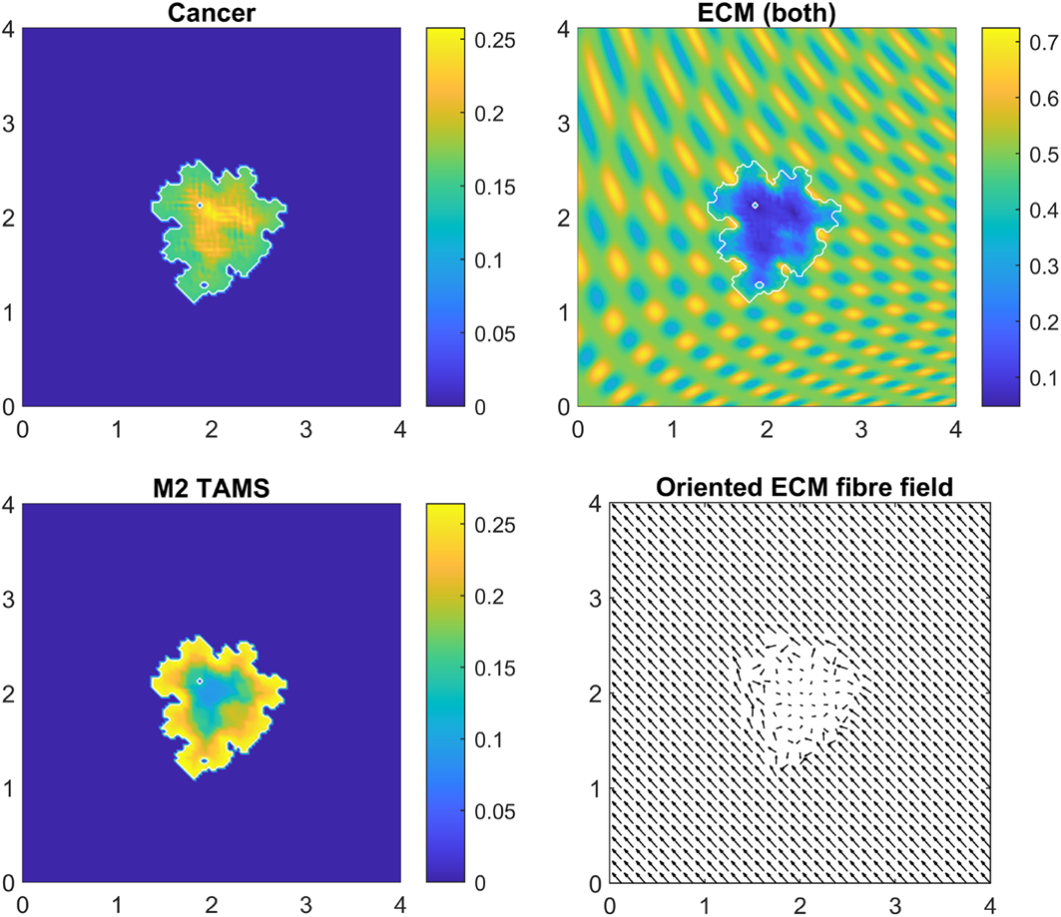
Baseline simulation at time $50 \Delta t$ where none of the newly introduced adhesions is present i.e. we set ${\mathbf {S}}_\mathrm{cM} = 0$, ${\mathbf {S}}_\mathrm{MM} = 0$, ${\mathbf {S}}_\mathrm{Mc} = 0$ and ${\mathbf {S}}_\mathrm{MF} = 0$

In Fig. 8, we show the distribution of macroscopic variables at time $50 \Delta t$, when we assume that all macrophage adhesion terms are zero: ${\mathbf {S}}_\mathrm{cM} = 0$, ${\mathbf {S}}_\mathrm{MM} = 0$, ${\mathbf {S}}_\mathrm{Mc} = 0$ and ${\mathbf {S}}_\mathrm{MF} = 0$ (while all other parameters are as in Appendix A). We observe that in this case the M2 TAMs are located near the outer boundary of the tumour. This is the result of the assumptions that macrophages infiltrate the tumour though the outer boundary (see Eq. (10)) and that macrophages diffuse with constant coefficient. Further, we observe that the initial homogeneous cancer cell density becomes heterogeneous due to the many cancer cell adhesion processes, highly influenced by the rearranged and degraded ECM (caused by both cancer cells and TAMs). On the other hand, in peritumoral regions, the ECM degradation creates free space for the tumour to expand and spread to the neighbouring tissues resulting in some *tumour fingering*, which gives an irregular tumour domain (that mainly follows the ECM pattern). This effect is complemented by the rearrangement of the micro-fibres that ultimately induces new a fibre structure. For illustrative purposes, in Fig. 8, we coarsen fourfold the ECM fibre field.

*The Impact of Macrophage Interactions on Tumour Dynamics*

**Fig. 9:**
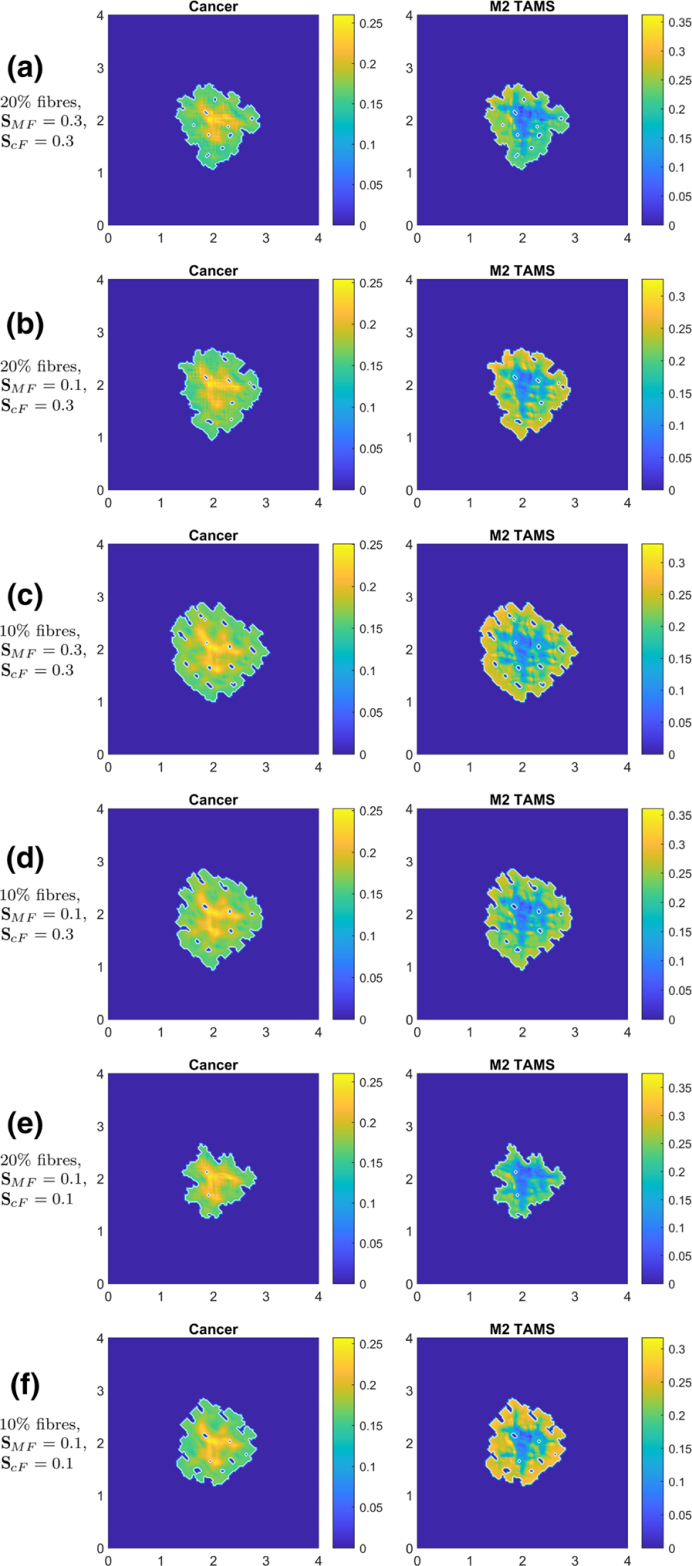
Simulations illustrating the effects of each freshly introduced adhesion separately. Hence, **a** corresponds to the baseline solution (also seen in Fig. 8), and **b**–**e** represent the effect of each strength ${\mathbf {S}}_\mathrm{cM}$, ${\mathbf {S}}_\mathrm{MM}$, ${\mathbf {S}}_\mathrm{Mc}$ and ${\mathbf {S}}_\mathrm{MF}$, respectively. Finally, **f** corresponds to their combined effects (i.e., we take ${\mathbf {S}}_\mathrm{cM}$, ${\mathbf {S}}_\mathrm{MM}$, ${\mathbf {S}}_\mathrm{Mc}$ and ${\mathbf {S}}_\mathrm{MF}$ from the parameter set ${\mathcal {S}}$ in “Appendix A”). Each simulation presented in this figure uses the initial conditions from (21), and Fig. 7. Moreover, each simulation corresponds to $50 \Delta t$

In Fig. 9, we investigate the effect of each individual adhesion interaction that we introduced in this paper i.e., cancer–M2, M2–M2, M2–cancer and M2–fibres interactions. For comparison purposes, Fig. 9a shows again the baseline cancer and TAM dynamics (as copied from Fig. 8).

In Fig. 9b, we show the effect of cancer–M2 adhesion interaction (i.e. ${\mathbf {S}}_\mathrm{cM} = 0.125$, while ${\mathbf {S}}_\mathrm{MM}={\mathbf {S}}_\mathrm{Mc}={\mathbf {S}}_\mathrm{MF}=0$). Interestingly, we do not see much difference compared to Fig. 9a (baseline simulation). This may indicate that this particular interaction is overwhelmed by the many other cancer cell–cell and cell–ECM adhesive interactions, and thus the cancer–M2 adhesion alone is not powerful enough to lead to a distinct tumour invasion pattern.

In Fig. 9c, we show the effect of M2 TAMs self-adhesion (i.e. ${\mathbf {S}}_\mathrm{MM} = 0.175$ and ${\mathbf {S}}_\mathrm{cM}={\mathbf {S}}_\mathrm{Mc}={\mathbf {S}}_\mathrm{MF}=0$). Compared to the baseline simulations, here the density of M2 TAMs becomes higher in the peripheral tumour region. As expected, when macrophages infiltrate the tumour, they prefer not to migrate but to stay together (due to ${\mathbf {S}}_\mathrm{MM}>0$).

In Fig. 9d, we consider the M2–cancer adhesion process (i.e. $S_\mathrm{Mc} = 0.125$, and ${\mathbf {S}}_\mathrm{cM}={\mathbf {S}}_\mathrm{MM}={\mathbf {S}}_\mathrm{MF}=0$). We notice here a more aggressive tumour fingering morphology compared to the baseline result. Moreover, the minimum of the M2 TAMs density inside the tumour domain is also decreased. To understand the reason behind this, we refer to the M2–cancer adhesion part of (12), where we note that this process does not only depend on the cancer cell density but also on the free space available (accounted for via $(1-\rho (\mathbf{u }))^{+}$). Therefore, M2 TAMs prefer areas of the tumour domain where the density of the cancer cells is high yet not too high, so that there is some free space available. Hence, this process could be one of the mechanisms responsible for accumulating and keeping M2 TAMs in the peripheral region.

In Fig. 9e, we consider the effect of M2–fibre adhesion (to explore the possibility that M2 TAMs movement depends also on the oriented ECM fibres). To this end, we set ${\mathbf {S}}_\mathrm{MF} = 0.3$ (and ${\mathbf {S}}_\mathrm{cM}={\mathbf {S}}_\mathrm{MM}={\mathbf {S}}_\mathrm{Mc}=0$). Comparing this simulation result with the baseline result shown in Fig. 9a, we can see that the M2 TAMs follow the fibre orientations. Since we assumed aligned ECM fibres (i.e., induced by the oriented ECM fibre density shown in Fig. 7b), the M2 TAM density in the peripheral region is higher in the direction of the oriented ECM fibres (as the initial ECM fibres have all the same top-left orientations on the macro-scale), and so at the top-left region of the tumour the M2 TAMs macrophages density is higher. This indicates that M2 TAMs accumulations may also depend on the oriented ECM fibre distribution.

Finally, in Fig. 9f, we combine all these adhesion processes (i.e. we use the values from the parameter set ${\mathcal {S}}$ in “Appendix A” for ${\mathbf {S}}_\mathrm{cM}$, ${\mathbf {S}}_\mathrm{MM}$, ${\mathbf {S}}_\mathrm{Mc}$ and ${\mathbf {S}}_\mathrm{MF}$). Until this point, we have not emphasised the fact that by considering each of these processes separately, we observed a slightly smaller tumour spread compared to the baseline simulation. However, as shown in Fig. 9f, by combining all these adhesion processes, we see an increase in tumour spread. This suggests that it may not be enough to focus only on one aspect of macrophages and rather we need to focus on these processes as a whole in order to stop the pro-tumoural behaviour of the M2 TAMs. Hence, these different adhesion processes may work with and magnify each other in an underlying fashion that creates a favourable environment for tumour development.

*The Impact of ECM Fibre Structure and Fibre-Adhesion Strengths on Tumour Dynamics*

**Fig. 10:**
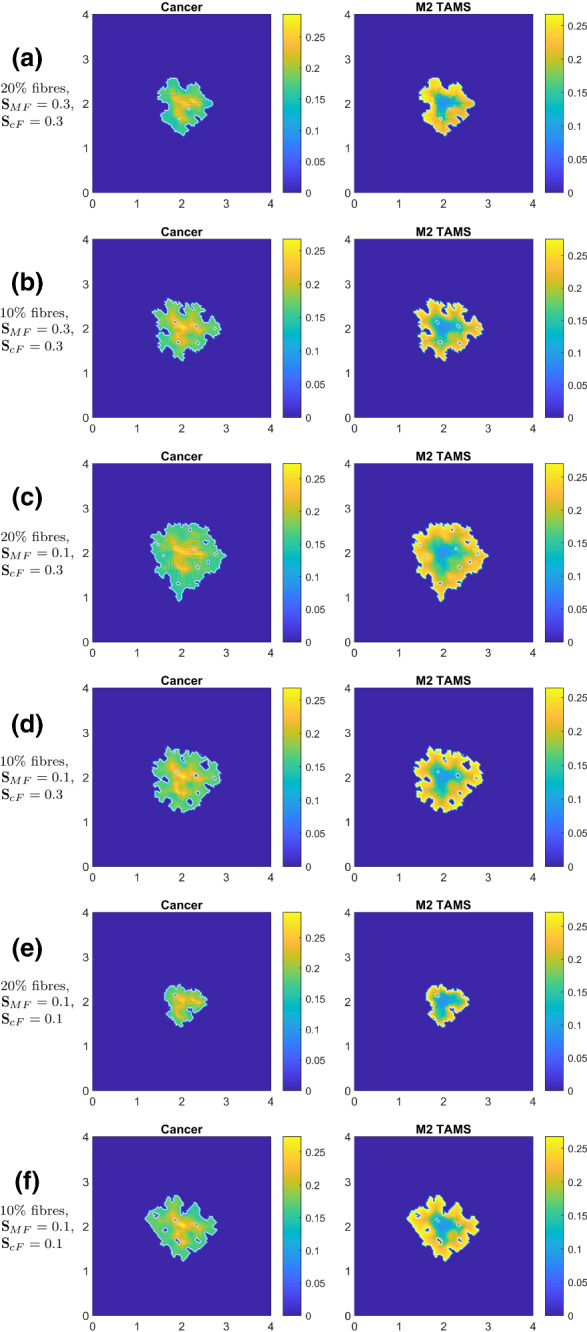
Simulations when only M2–fibre adhesion is present (i.e., we set ${\mathbf {S}}_\mathrm{cM} = 0$, ${\mathbf {S}}_\mathrm{MM} = 0$, ${\mathbf {S}}_\mathrm{Mc} = 0$) at time $50 \Delta t$: **a** baseline for this figure and so it is identical to Fig. 9e with $20\%$ fibres ($80\%$ non-fibre ECM) and ${\mathbf {S}}_\mathrm{cF} = 0.3$, ${\mathbf {S}}_\mathrm{MF} = 0.3$; **b** same fibre-adhesion strengths but with only $10\%$ fibres ($90\%$ non-fibre ECM); **c** $20\%$ fibres with decreased M2–fibre adhesion ${\mathbf {S}}_\mathrm{MF} = 0.1$; **d** $10\%$ fibres with decreased M2–fibre adhesion ${\mathbf {S}}_\mathrm{MF} = 0.1$; **e** $20\%$ fibres and both cancer and M2–fibre adhesion strengths are reduced ${\mathbf {S}}_\mathrm{cF} = 0.1$ and ${\mathbf {S}}_\mathrm{MF} = 0.1$; **f** $10\%$ fibres with the reduced fibre adhesion strengths ${\mathbf {S}}_\mathrm{cF} = 0.1$ and ${\mathbf {S}}_\mathrm{MF} = 0.1$

Next, we explore how the fibrous composition of ECM and the corresponding fibre-adhesion strengths affect the evolution of the solid tumour. In Fig. 10, we investigate the effect of changing (i) the ECM fibre percentage (compared to the non-fibre ECM) and (ii) the M2–fibre and cancer–fibre adhesion strengths, for the particular case where we have only M2–fibre adhesion (i.e. ${\mathbf {S}}_\mathrm{cM} = 0$, ${\mathbf {S}}_\mathrm{MM} = 0$, ${\mathbf {S}}_\mathrm{Mc} = 0$). For comparison purposes, in Fig. 10a we present again the M2–fibre adhesion case (i.e., the one shown in Fig. 9e, where ${\mathbf {S}}_\mathrm{MF} = 0.3$, and ${\mathbf {S}}_\mathrm{cF} = 0.3$.

In Fig. 10b, we decrease the fibre magnitude and take the ratio between the fibre and non-fibre ECM to be $10\%$:$90\%$. In this case, we see both more tumour fingers and an increase in the tumour spread. This is expected since by decreasing the fibre percentage, we also decrease the fibre adhesions. Moreover, the accumulation of fibres at tumour boundary becomes less dense, allowing the tumour to spread further, more efficiently.

In Fig. 10c, we consider again the baseline ECM fibre percentage ($20\%$) but we decrease the M2–fibre adhesion strength to ${\mathbf {S}}_\mathrm{MF} = 0.1$ (while keeping the cancer–fibre adhesion strength ${\mathbf {S}}_\mathrm{MF} = 0.3$). We observe here that due to the changes within the oriented ECM fibres distribution (caused by the fibres rearrangement at the micro-scale induced by the macro-scale spatial flux of cancer cells and M2 TAMs), the macrophages cannot move efficiently within the tumour micro-environment, and within the assumed conditions of low macrophages–fibre adhesion strength, the effect of the macrophages diffusion increases. Comparing these simulation results with those in Fig. 10a, we see a considerable increase in tumour spread. This suggests that the decrease in the M2–fibres adhesion leads to a diffusion-dominated macrophage movement, which likely helps tumour spread indirectly via the degradation of ECM.

In Fig. 10d, we present the simulation results when we decrease the fibre percentage to 10% and keep a low small M2–fibre adhesion strength (${\mathbf {S}}_\mathrm{MF} = 0.1$, as in Fig. 10c. Compared to the situation addressed in Fig. 10b, where we had larger macrophage-fibre adhesion strength, we note here an increase in the tumour spread. However comparing now the results in row 4 to the ones in Fig. 10c (where we had identical adhesion strength, but with increased level of ECM fibres to $20\%$ was considered) we observe a decrease in the tumour spread. Hence, the decreased level of macrophages–fibres adhesion strength (considered in simulations on rows 3 and 4) reverses the observation concerning tumour spread that emerged by comparing Fig. 10a, b (with a higher level of macrophages–fibres adhesion strength, ${\mathbf {S}}_\mathrm{MF}=0.3$). Hence, macrophages–fibres-mediated movement exerts a level of control on the tumour spread and the evolution of its morphology, by monotonically reducing tumour diffusive spread and at the same time stimulating lobular cancer invasion patterns.

In Fig. 10e, f, we investigate the effects of weak M2–fibre adhesion combined with weak cancer–fibre adhesion (i.e. ${\mathbf {S}}_\mathrm{cF} = 0.1$, ${\mathbf {S}}_\mathrm{MF} = 0.1$). Comparing the simulation results in Fig. 10e with those in Fig. 10c, we conclude that by reducing the cancer–fibre adhesion strength, we decrease significantly the tumour spread. However, if we reduce also the amount of fibres to 10% (see rows 5 and 6), we see an increase in tumour spread (likely due to larger cell–non-fibre adhesions).

**Fig. 11:**
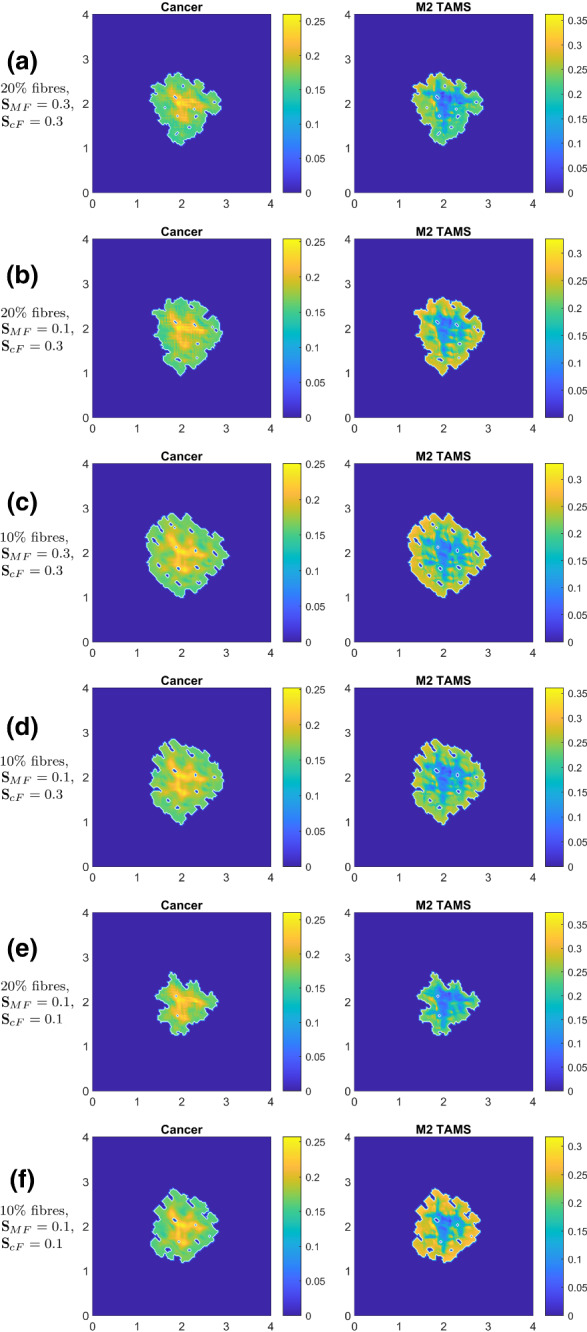
Simulations at time $50 \Delta t$ when all new M2 TAM adhesions are present: i.e., using the values from the parameter set ${\mathcal {S}}$ (see “Appendix A”, for ${\mathbf {S}}_\mathrm{cM}$, ${\mathbf {S}}_\mathrm{MM}$, ${\mathbf {S}}_\mathrm{Mc}$) while altering ${\mathbf {S}}_\mathrm{cF}$ and ${\mathbf {S}}_\mathrm{MF}$: **a** the baseline for this figure that is identical to Fig. 9f); **b** decreased M2–fibre adhesion ${\mathbf {S}}_\mathrm{MF} = 0.1$ with $20\%$ fibres; **c** $10\%$ fibres with ${\mathbf {S}}_\mathrm{MF} = 0.3$; **d** $10\%$ fibres with decreased M2–fibre adhesion ${\mathbf {S}}_\mathrm{MF} = 0.1$; **e** $20\%$ fibres and both cancer and M2–fibre adhesion strengths are reduced ${\mathbf {S}}_\mathrm{cF} = 0.1$ and ${\mathbf {S}}_\mathrm{MF} = 0.1$; **f** $10\%$ fibres with the reduced fibre adhesion strengths ${\mathbf {S}}_\mathrm{cF} = 0.1$ and ${\mathbf {S}}_\mathrm{MF} = 0.1$

Next, in contrast to Fig. 10, where no macrophage–macrophage self-adhesion nor cancer–macrophage or macrophage–cancer adhesion relations were considered, we address the case in which these processes are present, namely ${\mathbf {S}}_\mathrm{cM}>0$, ${\mathbf {S}}_\mathrm{MM}>0$ and ${\mathbf {S}}_\mathrm{Mc}>0$ (with the baseline values given in “Appendix A”, used also in Fig. 9f). Furthermore, as before, we vary the values of cell–fibres and macrophages–fibre adhesion, ${\mathbf {S}}_\mathrm{cF}$ and ${\mathbf {S}}_\mathrm{MF}$. In these conditions, in Fig. 11, we explore the importance of the M2–fibre adhesion compared to the rest of the adhesion terms. Again, for comparison purposes, in Fig. 11a we show the baseline dynamics of this case, as copied from Fig. 9f.

In these new conditions, Fig. 11a–d shows that changes in macrophage–fibre adhesion at ECM fibres regimes of both 20$\%$ and 10$\%$ do not have a significant impact on the tumour spread (although a smaller cancer spread is noticed when lower $S_\mathrm{MF}$ is considered).

However, as shown in the ECM regime with $20\%$ fibres levels, the comparison of the results from row 5 (where low cancer–fibre adhesion ${\mathbf {S}}_\mathrm{cF} = 0.1$ is considered) with those in row 2 highlights the importance of cancer–fibres adhesion within the invasion, as we record higher tumour spread for higher values of ${\mathbf {S}}_\mathrm{cF}$. The same behaviour is observed through the comparison of row 4 and row 6, where the same variation of ${\mathbf {S}}_\mathrm{cF}$ when we consider a lower level of ECM fibres and of macrophage–fibre adhesion, ${\mathbf {S}}_\mathrm{MF}=0.1$.

Finally, by comparing Fig. 11a, b, e, we observe that it is the cell–fibres rather than the macrophages–fibre adhesion that plays the dominant role at higher level fibres ($20\%$) within the ECM. However, the comparison between rows 3, 4 and 6 of Fig. 11 shows that in regimes with lower levels of fibres ($10\%$) within the ECM, the influence of the macrophages–fibres adhesion on the overall cancer invasion is still important.

*The Effect of Random Micro-fibre Distribution*

In all our previous numerical simulations, we used the micro-fibre structure described in Fig. 7b that we repeated for each micro-fibre domain $\delta Y(x)$, thus inducing the same fibre orientation at each macro-scale node (top-left orientation seen in Fig. 8). Now we consider random mass distributions of micro-fibres for each micro-domain $\delta Y(x_{i},y_{j})$, (i.e. we draw five random straight lines in each $\delta Y$ rather than use the five-line configuration presented in Fig. 7b).

**Fig. 12:**
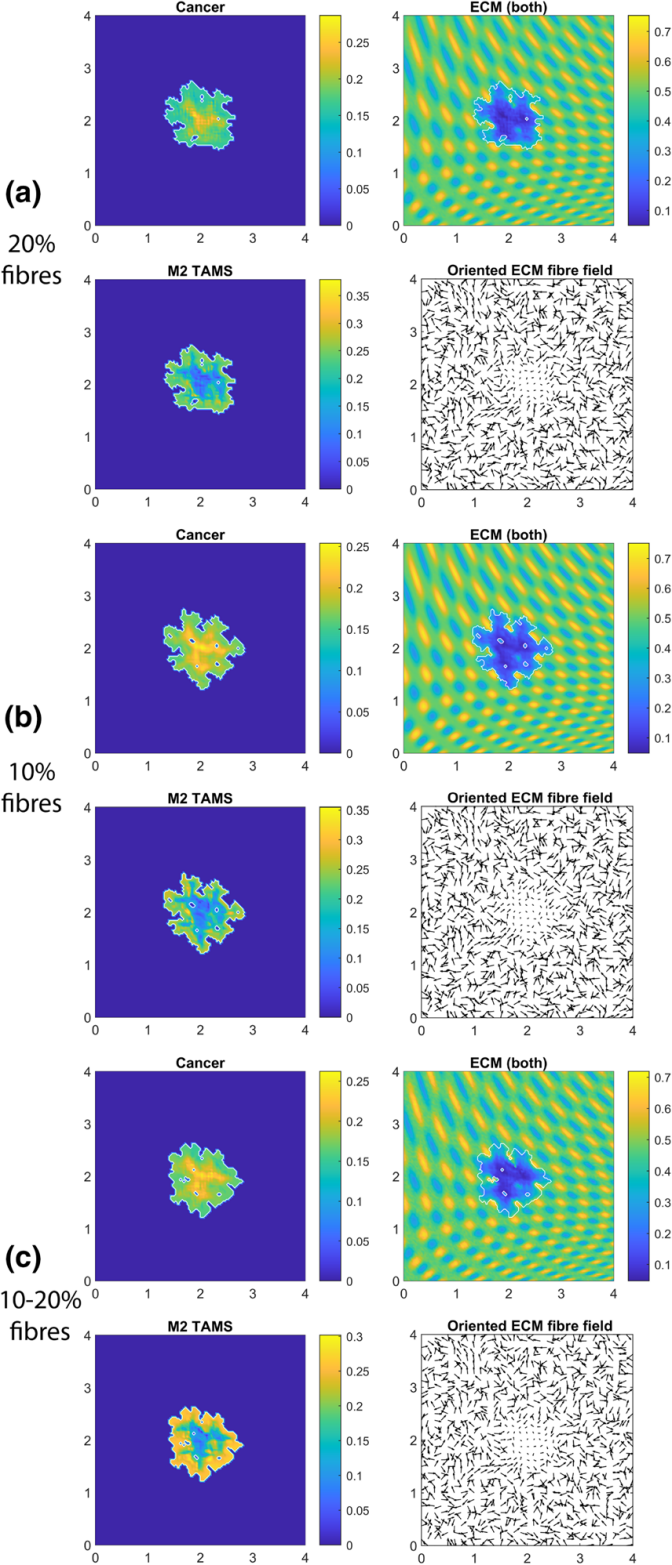
Simulations with random fibre structures for each micro-fibre domain $\delta Y$ combined with every adhesion terms (i.e., using ${\mathbf {S}}_\mathrm{cM}$, ${\mathbf {S}}_\mathrm{MM}$, ${\mathbf {S}}_\mathrm{Mc}$, ${\mathbf {S}}_\mathrm{MF}$ and ${\mathbf {S}}_\mathrm{cF}$ from the parameter set ${\mathcal {S}}$ “Appendix A”) at time $50 \Delta t$: **a** (first two rows) represents the result of random micro-fibre structure, but their macro-scale magnitude is still considered to be a constant $20\%$; **b** (third and fourth rows) presents the result of $10\%$ fibres with random structures; **c** (last two rows) also uses random structure but also uses random macro-scale magnitude for each micro-domain $\delta Y$ between $10\%$ and $20\%$

In Fig. 12, we present three simulations with randomised mass distributions of micro-fibres on each $\delta Y(x_{i},y_{j})$, $i, j\in \{1,\dots ,n\}$ (where the same random distribution is used for all three simulations as an initial condition). Here, we focus on the case when all of the adhesion terms are present (i.e. ${\mathbf {S}}_\mathrm{cM}$, ${\mathbf {S}}_\mathrm{MM}$, ${\mathbf {S}}_\mathrm{Mc}$, ${\mathbf {S}}_\mathrm{MF}$, ${\mathbf {S}}_\mathrm{cF}>0$, as given in “Appendix A”).

In Fig. 12a, we use $20\%$ fibres, and we observe that due to the random fibres, the tumour spread is reduced (by $\approx 25\%$) compared to Fig. 9f. This is not surprising as we moved from aligned fibres to a random oriented fibres, where the movement is expected to be slower.

In Fig. 12b, we reduce the fibres to $10\%$, and we observe an even greater tumour reduction compared to the aligned fibre case in Fig. 10b (approximately $43\%$ reduction in tumour area). Also, as before, we see an increase in the tumour spread when we decrease the fibres from $20\%$ to $10\%$.

In Fig. 12c, we not only consider random micro-fibre structures but also use random macro-fibre magnitudes (i.e. at each macro-node, we now have a random level of fibres between $10\%$ and $20\%$). For this case, we see that tumour spread is slightly greater than for the $20\%$ fibre case, but slightly smaller than for the $10\%$ case. We can conclude from here that using random fibres may considerably reduce tumour spread, but it does not introduce new tumour morphology.

### Different Tissue Conditions

Based on biological knowledge (Hanahan and Weinberg 2011; Weinberg 2006), given the complex and naturally multiscale cancer invasion process, a solid cancer progresses further within the surrounding tissue provided that significant but not complete tumour peritumoural ECM degradation will have been achieved, i.e., favourable tissue conditions for invasion are met. In all the simulations that we carried out so far in this work, these tissue conditions for tumour progression have been explored within the framework defined in Trucu et al. (2013), where these were captured through a tissue parameter $\beta \in (0,1)$, which characterises the relative level of significant degradation of the peritumoural ECM. For all the results presented in Figs. 8, 9, 10, 11, 12, this parameter was set to the level of $\beta :=0.65$, which corresponds to relatively mild conditions for invasion. However, as we wish to explore our modelling also for more demanding tissue conditions for tumour invasion that requires more elevated levels of significant peritumoural ECM degradation, we now vary the parameter $\beta$ by increasing its value.

**Fig. 13:**
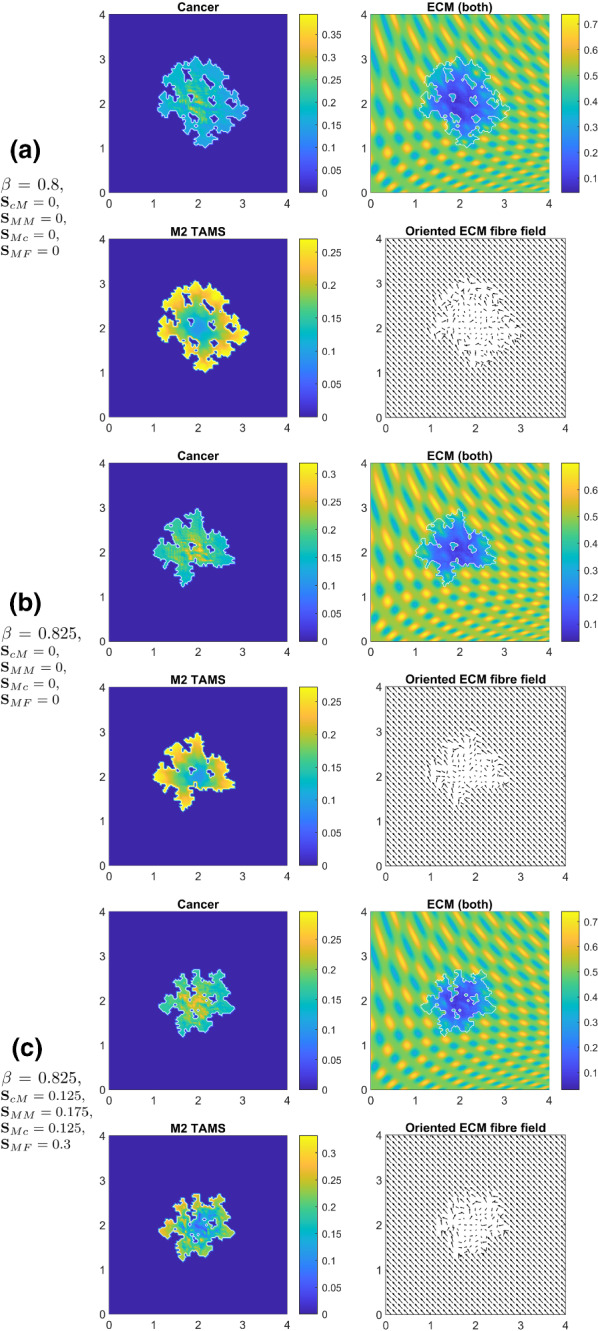
Simulations, at time $50 \Delta t$, with the extended model (14) for different tissue conditions ($\beta$), while we consider $20\%$ fibres. **a** simulation with zero-macrophage adhesion coefficients (i.e. ${\mathbf {S}}_\mathrm{cM} = 0$, ${\mathbf {S}}_\mathrm{MM} = 0$, ${\mathbf {S}}_\mathrm{Mc} = 0$ and ${\mathbf {S}}_\mathrm{MF} = 0$) and an increased $\beta = 0.8$; **b** the tissue parameter is further increased to $\beta = 0.825$ without considering any macrophage adhesion processes (i.e. ${\mathbf {S}}_\mathrm{cM} = 0$, ${\mathbf {S}}_\mathrm{MM} = 0$, ${\mathbf {S}}_\mathrm{Mc} = 0$ and ${\mathbf {S}}_\mathrm{MF} = 0$); **c** simulation with $\beta = 0.825$ where we take the values for ${\mathbf {S}}_\mathrm{cM}$, ${\mathbf {S}}_\mathrm{MM}$, ${\mathbf {S}}_\mathrm{Mc}$ and ${\mathbf {S}}_\mathrm{MF}$ from the Parameter set ${\mathcal {S}}$ in “Appendix A”

To illustrate our results in new tissue conditions favourable for tumour progression induced by elevated values of $\beta$, in Fig. 13a, we show the result for our model without the new adhesion processes and tissue conditions induced by $\beta = 0.8$. Here, we observe a pronounced lobular formation and increased fingering within the tumour morphology where the number and size of the islands inside tumour are also increased. Then, the results in Fig. 13b show that, by increasing further the tissue parameter $\beta = 0.825$ (while keeping the same diffusion and adhesion regime), we obtain an even more aggressive tumour fingering and lobular formation, and a rather decreased tumour area compared to Fig. 13a. Finally, in Fig. 13c, we show the results that we obtain when we re-introduce the adhesion processes (i.e. we take ${\mathbf {S}}_\mathrm{cM}$, ${\mathbf {S}}_\mathrm{MM}$, ${\mathbf {S}}_\mathrm{Mc}$ and ${\mathbf {S}}_\mathrm{MF}$ from the Parameter set ${\mathcal {S}}$ in “Appendix A”) which results in further decrease in the tumour area while maintaining a significant tumour fingering and lobular behaviour.

## Conclusion

In this study, we further developed the multiscale moving-boundary framework for tumour invasion introduced in Shuttleworth and Trucu (2019), Trucu et al. (2013), by including also a tumour-associated macrophage cell population, with the goal of investigating the interactions between macrophages directional movement and the directionality of ECM fibres on the overall tumour spread. We focused on the M2-like macrophages since various experimental and clinical studies have shown that these cells are not only one of the most abundant immune cell populations that infiltrate the tumour mass (Kelly et al. 1988; Vinogradov et al. 2014), but they are also involved in the degradation of ECM (Dollery and Libby 2006; Madsen et al. 2013; Newby 2008; Rath et al. 2019) thus helping tumour invasion. We considered this modelling and computational approach since in the experimental literature there is still little knowledge about the directional interactions of macrophages with the directional/random ECM architecture, and how this affects tumour spread.

To address this lack of knowledge and to propose new hypotheses on these interactions, we started with the modelling framework in Shuttleworth and Trucu (2019) that focused on the dynamics of tumour cells and extracellular matrix at both micro- and macro-scales and introduced a new macro-scale equation for the pro-tumour M2 cells. In this new equation, we considered the M2 TAMs movement to be both random and directed, with the directed movement being the result of self-adhesion and fibre adhesions, as well as M2–cancer adhesion. The rest of the M2 TAMs dynamics included a linear death term, a proliferation term and an influx term where we assumed a constant influx of M2 TAMs on the outer tumour boundary. Since the M2 TAMs secrete MDEs, the degradation of both ECM components is not only influenced by the density of the cancer cells but also by the density of M2 TAMs. Therefore, the macrophages have a direct contribution to the source for the proteolytic micro-dynamics of MDEs occurring at the invasive edge of the tumour, which ultimately determines the way the tumour boundary is relocated during invasion. Furthermore, as macrophages are moving, they influence the re-arrangement of the micro-fibres through the flux that they induce. Thus, the M2 macrophages are involved in both the *top-down* and *bottom-up* links of the two interconnected multiscale subsystems that take place both at leading edge and on the bulk of an invading tumour (which are schematically summarised in Fig. 6).

While the genuine heterogeneous and multiphase structure of the ECM has been acknowledged by the entire experimental and biological community (Hanahan and Weinberg 2011; Hynes and Naba 2012), the two-phase ECM modelling perspective proposed in this current work (as well as in Shuttleworth and Trucu (2019, 2020a, 2020b)) not only that was naturally motivated, but the insights that this brings (through the possibility of exploring in detail the multiscale and complex adhesive interaction between the cells and macrophages on the one hand and both the non-fibres ECM phase (Ghosh et al. 2017; Gras 2009; Gras et al. 2008; Jacob et al. 2016) and the oriented ECM fibre phase (Wolf et al. 2009; Wolf and Friedl 2011)) opens the way for a deeper understanding of both directed and undirected tumour cell population movement in the presence of M2 TAMs macrophages. This will have direct implication in the planned future works on drug and chemotherapy delivery which naturally follows the non-fibres ECM phase, rather than the ECM fibres phase.

We used this new extended multiscale moving-boundary mathematical model to explore some biological hypotheses regarding the role of M2 cells on tumour spread. First, we investigated the individual effects of the cancer–M2 and M2–cancer adhesions, as well as the M2–self- and M2–fibre adhesions, and we concluded that individually these interactions do not lead to a significant increase in tumour spread. However, they do change the tumour morphology, by leading to various accumulation sites for the M2 TAMs (see Fig. 9). We also showed that combining all these different adhesion terms leads to an increase in tumour spread. Then, we explored how the fibrous component of the ECM and the corresponding fibre adhesion strengths could affect tumour development (see Figs. 10 and 11). The simulations showed that decreasing the fibre density could be helpful to reduce tumour spread, but this depended also on the M2 TAMs directed movement via fibre adhesion. We also investigated the importance of M2–fibre adhesion compared to the rest of the adhesions. In our model, this suggested that the M2–fibre adhesion plays a minor role and does not significantly affect tumour development. Moreover, we considered an unstructured, random micro-fibre structure that revealed that although it does not introduce new properties, it can reduce the spread quite dramatically. We note here that all these numerical results were obtained with a low $\beta$ value (to be consistent with the studies in Shuttleworth and Trucu (2019, 2020a, 2020b)), which means that even if the ECM was not significantly degraded, the tumour was still able to invade the surrounding tissue. Therefore, changes in the various parameter values considered in Figs. 9, 10, 11, 12 did not lead to huge differences in tumour invasion patterns. However, in Fig. 13, we present some simulations for the tumour dynamics , as we varied the tissue environment parameter $\beta$. We have seen that for higher $\beta$, changes in cell–cell and cell–matrix adhesion strengths lead to completely different tumour invasion patterns, with more tumour fingering, and clear unidirectional movement towards the direction of ECM fibres.

All these numerical results indicate that the combined effects of macrophage–tumour–ECM interactions (via different cell–cell and cell–fibre adhesions), and the constant-vs.-density-dependent diffusion, are important for tumour development. Moreover, these results allow us to conclude that it is difficult to ascertain at a macroscopic level the specific molecular (i.e. adhesion) mechanisms that could be responsible for the observed macroscopic patterns of tumour spread and/or accumulation.

The complexity of the interaction between tumour cell and macrophages exceeds the context captured in this work through the mutual adhesion terms involved in the macroscopic model equations. For example, this approach does not capture explicitly the complex cross talk between tumour cells and macrophages via chemokines. Such aspects will be investigated in future studies. Further, due to the complexity of the multiscale moving-boundary framework and the numerical simulations of these equations, in this work we focused only on the dynamics of the tumour and the immune cells infiltrating it. For this reason, we assumed that macrophages enter the tumour though tumour boundary. In the future, we will extend this modelling approach to account also for the dynamics of macrophages outside the tumour (which are attracted to the tumour site via chemokines secreted by the tumour cells (Green et al. 2009)).

Finally, another aspect that will be addressed in future work is the analysis of the complex multiscale numerical framework proposed in this study. As this computational framework is new, this is an open problem that requires a proper independent investigation.

## Figures and Tables

**Fig. 14:**
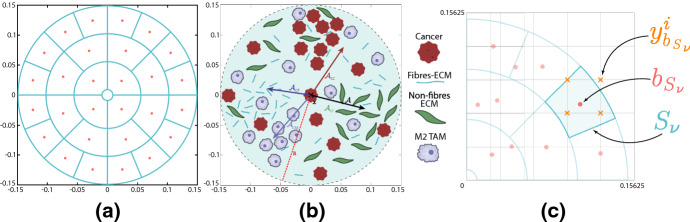
The sensing region *B*(0, *R*). **a** Decomposition of the region using annulus sectors $S_{\nu }$ with barycentres $b_{S_{\nu }}$, highlighted with red dots. **b** Illustration of the vector-valued ${\mathcal {A}}_{c}(x,y)$, influenced by the surrounding components and the strength of these bonds. **c** Highlight of an annulus sector $S_{\nu }$ with its barycentre $b_{S_{\nu }}$ as well as the four neighbouring on-grid points $\{y_{b_{S_{\nu }}}^i\}_{i=1,4}$

## Parameter Values

In Table 2, we present the baseline set of parameters used in this work.

**Table 2:** Parameter set ${\mathcal {S}}$

Variable	Value	Description	Reference
$D_{c}$	$10^{-4}$	Diffusion coeff. for the cancer cell population *c*	Domschke et al. (2014)
$D_{M}$	$5 \times 10^{-5}$	Diffusion coeff. for the M2 TAM population *M*	Estimated using Hayenga et al. (2015)
$D_{m}$	$2.5 \times 10^{-3}$	Diffusion coeff. for MDEs	Peng et al. (2017)
${\mathbf {S}}_{\max }$	0.5	Cell–cell adhesion coeff.	Shuttleworth and Trucu (2019)
${\mathbf {S}}_{\min }$	0.01	Minimum level of cell–cell adhesion	Estimated
${\mathbf {S}}_\mathrm{cl}$	0.01	Cell–non-fibre adhesion coeff.	Shuttleworth and Trucu (2019)
${\mathbf {S}}_\mathrm{cM}$	0.125	Cell–macrophage adhesion coeff.	Estimated
${\mathbf {S}}_\mathrm{cF}$	0.3	Cell–fibre adhesion coeff.	Domschke et al. (2014)
${\mathbf {S}}_\mathrm{MM}$	0.175	Macrophage self-adhesion coeff.	Estimated
${\mathbf {S}}_\mathrm{Mc}$	0.125	Macrophage–cancer adhesion coeff.	Estimated
${\mathbf {S}}_\mathrm{MF}$	0.3	Macrophage–fibre adhesion coeff.	Estimated
$\mu _{c}$	0.25	Proliferation coeff. for cancer cell population *c*	Domschke et al. (2014)
$\mu _\mathrm{cM}$	1.4	Coeff. for the M2 TAMs dependence in the	Estimated using Hu et al. (2015)
		cancer cell proliferation	
$M_{0}$	0.05	Influx of the M2 TAMs	Estimated
$\mu _{M}$	0.1	Inside tissue proliferation rate of M2 TAMs	Estimated
$\mu {MF}$	2.4	Coeff. for the ECM stiffness dependence in the	Estimated using Hayenga et al. (2015)
		M2 TAMs proliferation	
$d_{M}$	0.03	Decay rate of *M*	Estimated using Strachan et al. (2013)
$\lambda _\mathrm{lc}$	1.0	Degradation coeff. for non-fibre ECM due	Estimated
		to the tumour	
$\lambda _\mathrm{lM}$	1.0	Degradation coeff. for non-fibre ECM due to	Estimated
		the M2 TAMs	
$\gamma _\mathrm{Fc}$	0.75	Degradation coeff. for fibre ECM due to the	Estimated
		tumour	
$\gamma _\mathrm{FM}$	0.75	Degradation coeff. for fibre ECM due to	Estimated
		the M2 TAMs	
$\alpha _{1}$	0	Baseline remodelling	Estimated
$\alpha _{2}$	0	Remodelling of the non-fibre ECM due to the	Estimated
		M2 TAMs	
$\alpha _{c}$	1	MDE secreting rate by the cancer cells	Estimated
$\alpha _{M}$	1	MDE secreting rate by the M2 TAMs	Estimated
*R*	0.15	Sensing radius	Shuttleworth and Trucu (2019)
*r*	0.0016	Width of micro-fibres	Shuttleworth and Trucu (2019)
$f_{\max }$	0.6360	Maximum of micro-fibre density at any point	Shuttleworth and Trucu (2019)
*p*	0.2	Percentage of non-fibre ECM	Shuttleworth and Trucu (2019)
$h_{L}$	0.03125	Macro-scale spatial step-size	Trucu et al. (2013)
$\epsilon$	0.0625	Size of the boundary micro-domain $\epsilon Y (\mathbf{x })$	Trucu et al. (2013)
$\delta$	0.03125	Size of the fibre micro-domain $\delta Y (\mathbf{x })$	Shuttleworth and Trucu (2019)

## Definition of the Outer Boundary $\partial \varOmega _{o}(t)$

Let $x\in \partial \varOmega (t)$. Then, $x\in \partial \varOmega _{o}(t)$ if and only if there exists $\phi _{_{x}}:[0,\infty )\rightarrow {\mathbb {R}}^{d}$ such that the following four properties hold true simultaneously:

22 $$\begin{aligned} 1)\quad&\phi _{_{x}}(0)=x, \end{aligned}$$

23 $$\begin{aligned} 2)\quad&\phi _{_{x}}(s)\ne x, \quad \forall s\in (0, \infty ), \end{aligned}$$

24 $$\begin{aligned} 3)\quad&Im \phi _{_{x}}\setminus \{x\}\subset \complement \varOmega (t), \end{aligned}$$

25 $$\begin{aligned} 4)\quad&\lim \limits _{s \rightarrow \infty }dist (\phi (s), \partial \varOmega (t))=\infty , \end{aligned}$$

where $\forall \, s \in (0,\infty )$, we have $dist(\phi (s), \partial \varOmega (t)):=\inf \limits _{z\in \partial \varOmega (t)}\parallel \phi (s)-z \parallel _{_{2}}$ and represents the Euclidean distance from $\phi (s)$ to $\partial \varOmega (t)$.

## The Compact Support Function $\psi$

The smooth compact support function $\psi :{\mathbb {R}}^{d}\rightarrow {\mathbb {R}}_{+}$ used for the construction of standard mollifiers used in this paper is defined as usual by

26 $$\begin{aligned} \psi (x) := {\left\{ \begin{array}{ll} \dfrac{\exp \bigg ( \dfrac{1}{\parallel x \parallel _{2}^{2} - 1} \bigg )}{ \int \limits _{ {\mathbf {B}}(0, 1) } \exp \bigg ( \dfrac{1}{\parallel z \parallel _{2}^{2} - 1} \bigg ) dz}&{} \text {if } x \in {\mathbf {B}}(0, 1), \\ 0 &{} \text {if } x \notin {\mathbf {B}}(0, 1). \end{array}\right. } \end{aligned}$$

## Hadamard Product and Frobenius Inner Product

The Hadamard product of two square matrices *A* and *B* of the same dimensions is the matrix entry-wise product that is defined by

$$\begin{aligned} (A \circ B)_{i,j} = A_{i,j} \cdot B_{i,j}, \end{aligned}$$

which we use to derive our numerical scheme.

The Frobenius norm of an *n* by *m* matrix *A* is the square root of the sum of all elements of *A*, i.e.

$$\begin{aligned} \sum (A) := \text {Tr} \big ( A^H A \big ), \end{aligned}$$

where $A^H$ is the conjugate transpose.

## Details on the Fibre Relocation Vector and Micro-Fibre Rearrangement Process

As we mentioned in Sect. 2.2.2, the rearrangement process of the ECM fibres occurs on each micro-domain $\delta Y(x)$ via the spatial redistribution of the micro-fibres *f*(*z*, *t*). This process is initiated by a macroscopic cell population flux that gets formed at $(x,t)\in \varOmega (t)\times [0,T]$ (which accounts for both cancer cell and M2 TAMs fluxes) and acts on the mass distribution of micro-fibres on $\delta Y(x)$, causing the redistribution of these on both $\delta Y(x)$ and its neighbouring micro-domains. As detailed in Shuttleworth and Trucu (2019), this is done via an emerging relocation vector $\nu _{\delta Y(z)}$ that accounts not only for the rearrangement vector $r(\delta Y(x), t)$, but also upon the degree of alignment between the barycentral position vector $x_\mathrm{dir}(z):=z-x$ and the acting rearrangement vector $r(\delta Y(x), t)$ as well as both the level of fibres saturation at *z* and the level of occupancy at the target position $z^{*}$. Thus, the relocation vector $\nu _{\delta Y(z)}(z,t)$ is mathematically formulated as:

$$\begin{aligned} \nu _{\delta Y(x)}(z,t) := \big [ x_\mathrm{dir}(z) + r(\delta Y(x), t) \big ] \cdot \dfrac{f(z, t) [f_{\max } - f(z, t)]}{f^{*} + \parallel r(\delta Y(x), t) - x_\mathrm{dir}(z) \parallel _{2}} \cdot \chi _{ \{ f(\cdot , t)>0 \} }, \end{aligned}$$

with $x_\mathrm{dir}(z):=z-x$ denoting the associated barycentric position vector, $f_{\max }$ being the maximum level of fibres at any micro-location $\zeta \in \delta Y(x)$, $f^{*} = f(z, t)/f_{\max }$ being the microfibres level of saturation, and $\chi _{ \{ f(\cdot , t)>0 \} }$ representing the characteristic function of the micro-fibres support. To highlight their roles in the micro-scale process, we refer the reader again to Fig. 4, where we consider a typical example of $x_\mathrm{dir}(z)$, $r(\delta Y(x), t)$ and $\nu _{\delta Y(x)}(z,t)$.

Finally, the amount of fibres distributed at *z* that will be moved to the new micro-position $z^{*}$ is controlled by a movement probability $p_{_\mathrm{move}}$ that explores the fibre capacity available at the new location, and so this is given as

$$\begin{aligned} p_{_\mathrm{move}}:=\max \Bigg ( 0, \frac{f_{_{\max }}-f(z^{*})}{f_{_{\max }}} \Bigg ), \end{aligned}$$

the amount of fibres moving to $z^{*}$ is given by $p_\mathrm{move} \cdot f(z, t)$ and the rest of the fibres $(1 - p_\mathrm{move}) \cdot f(z, t)$ remain at position *z*.

## Brief Details of the Boundary Micro-Scale

As we mentioned in Sect. 2.2.3, to account for the proteolytic molecular processes occurring along the invasive edge of the tumour, we follow Trucu et al. (2013). To this end, we consider a time-dependent bundle of overlapping micro-domains ${\mathcal {P}}(t):=\{\epsilon Y\}_{\epsilon Y \in {\mathcal {P}}(t)}$ of appropriate cell-scale size $\epsilon$, as illustrated in Fig. 5, which cover the entire cell-scale neighbourhood of the tumour interface where the MDEs micro-dynamics takes place, i.e. $\partial \varOmega (t)\subset \cup _{\epsilon Y\in {\mathcal {P}}(t)}\epsilon Y$. This bundle of micro-domains ${\mathcal {P}}(t)$ enables us to decompose the micro-dynamics taking place on $\cup _{\epsilon Y\in {\mathcal {P}}(t)}\epsilon Y$, and to explore this on a union of micro-processes taking place on each micro-domain $\epsilon Y\in {\mathcal {P}}(t)$. Therefore, under the presence of the MDEs source induced by the macro-dynamics and defined in (19), as the MDEs are only assumed here to exercise a random movement within their molecular range (that dictates the size of our cell-scale $\epsilon >0$), their spatio-temporal evolution over $\epsilon Y$ during the time interval $[t_{0},t_{0}+\Delta t]$ is given by

27 $$\begin{aligned} \dfrac{\partial m}{\partial t} = D_{m}\Delta m + h(y, \tau ) \end{aligned}$$

where $$ D_{m}$$ is a constant diffusion coefficient. As we do not assume any memory from previous proteolytic activity, we always consider zero initial conditions. Further, as we assume that all the molecular activity occurs within $$\epsilon Y$$ as well as that there is no molecular transport over the boundary, we impose zero-flux boundary conditions.

Using the solution of (27), we adopt the technique developed in Trucu et al. (2013) to determine the relocation of the boundary. To that end, as described in Trucu et al. (2013), we select an appropriate dyadic decomposition $\{{\mathcal {D}}_{k}\}_{k\in {\mathcal {J}}}$ of the boundary micro-domain $\epsilon Y(x) \in {\mathcal {P}}(t)$. Furthermore, we denote by $y_{k}$ the barycentre of each ${\mathcal {D}}_{k}$ and we further select a subfamily of dyadic cubes $\{{\mathcal {D}}_{k}\}_{{\mathcal {J}}^{*}}$ that are furthest away from *x* and are outside of $\varOmega (t_{0})$ with the property that they still support a level of MDEs that exceeds the mean of MDEs distributed on $\epsilon Y(x)$. Then, the transitional measure of movement that was introduced in Trucu et al. (2013) (assessing the likelihood of the boundary to migrate based on the amount of MDEs transported in the peritumoural region) is given by

28 $$\begin{aligned} q(x) := \dfrac{\int \nolimits _{\epsilon Y (x) \setminus \varOmega (t_{0})} m(y, \tau _{f}) \; dy}{\int \nolimits _{\epsilon Y (x)} m(y, \tau _{f}) \; dy} \end{aligned}$$

and is used to evaluate whether the boundary point *x* will be moved to a new location or will stay at the same position. This assessment is carried out by comparing the likelihood for invasion given by the transitional measure of movement with respect to the significant level of peritumoural ECM degradation that is explored by a tissue parameter $\beta \in (0,1)$, as defined in Trucu et al. (2013). This tissue parameter $\beta$ explores the necessary level of significant (but not complete) ECM degradation for the tumour to progress and captures the tissue conditions for invasion through the tissue thresholds $\omega : (0,1) \times \epsilon Y (x_{B}) \rightarrow [0,1]$ given by

29 $$\begin{aligned} \omega (\beta , \epsilon Y(x)) := {\left\{ \begin{array}{ll} \sin \bigg [ \dfrac{\pi }{2} \bigg ( 1 - \dfrac{\alpha (x)}{\beta } \bigg ) \bigg ] &{} \text {if } \alpha (x) \le \beta , \\ \sin \bigg [ \dfrac{\pi ( \alpha (x) - \beta )}{2(1-\beta )} \bigg ] &{} \text {if } \alpha (x) > \beta , \end{array}\right. } \end{aligned}$$

where $\alpha (x)$ is defined by

$$\begin{aligned} \alpha (x) := \dfrac{l(x, t+\Delta t) + F(x, t+\Delta t)}{\sup \nolimits _{\xi \in \partial \varOmega (t_{0})} \big [ l(\xi , t+\Delta t) + F(\xi , t+\Delta t) \big ]}. \end{aligned}$$

Provided that sufficient degradation but not total destruction of the ECM has taken place over the time interval $[t_{0}, t_{0}+\Delta t]$, situation that is explored by the condition

$$\begin{aligned} q(x) > \omega (\beta , \epsilon Y (x_{B})), \end{aligned}$$

then the predicted tumour boundary movement is exercised in the direction $\eta _{\epsilon Y (x)}$ and magnitude $\xi _{\epsilon Y (x)}$ obtained from the boundary micro-dynamics (as described in Trucu et al. (2013)), given by

30 $$\begin{aligned} \begin{aligned} \eta _{\epsilon Y (x)}&{:=}\, x + \nu \sum _{l \in {\mathcal {J}}^{*}} \Bigg ( \int \limits _{{\mathcal {D}}_{l}} m(y, \tau _{f}) \; dy \Bigg ) \Big ( y_{l} - x \Big ), \qquad \nu \in [0, \infty ), \\ \xi _{\epsilon Y (x)}&{:=}\, \sum _{l \in {\mathcal {J}}^{*}} \dfrac{\int \limits _{{\mathcal {D}}_{l}} m(y, \tau _{f}) \; dy}{\sum \limits _{l \in {\mathcal {J}}^{*}} \int \limits _{{\mathcal {D}}_{l}} m(y, \tau _{f}) \; dy} \parallel \overrightarrow{x y_{l}} \parallel _{_{2}}. \end{aligned} \end{aligned}$$

This way, over the time interval $[t_{0}, t_{0}+\Delta t]$, the macroscopic tumour $\varOmega (t_{0})$ will be progressed into a newly expanded invasive tumour domain $\varOmega (t_{0}+\Delta t)$.

## Numerical Schemes

To describe our numerical approach, let us introduce some basic notation that will be used throughout the whole section. Let *L* be the length of *Y* and $h_{L} = \Delta x = \Delta y$ to be the spatial step size that is uniform in both direction. The uniform discretisation of the maximal tissue cube *Y* is represented through the set of discretised macro-spatial locations $\{(x_{i}, y_{j})\}_{i,j=1\ldots N}$, with $N=L/h_{L}+1$.

To numerically solve the macro-dynamics (14), we adopt the non-local predictor–corrector scheme introduced in Shuttleworth and Trucu (2019) (involving the Euler method as predictor, and a non-local Trapezoidal rule as corrector) and we follow Domschke et al. (2014), Gerisch and Chaplain (2006, 2008) to construct a flux limiter method for the spatial derivatives. Also, to evaluate the adhesion terms ${\mathcal {A}}_{c}$ and ${\mathcal {A}}_{M}$ defined in (5) and (12), respectively, we aim to use a fast convolution-driven approach. Finally, we detail the MDE-micro-scale calculations.

### Macro-Scale Dynamics

In this section, we focus on deriving the numerical scheme for model (14). Hence, in (14), we have two distinct spatial operators, namely a diffusion and an adhesion term. Since the form of both of these terms is similar for cancer cells and M2 TAMs, in the following, we will focus only on describing the scheme that we derive for the cancer cell population.

Focusing now on the discretisation of the spatial operator in (14), which can be expressed as

$$\begin{aligned} \nabla \cdot {\mathcal {F}}_{c}:=\nabla \cdot [(D^{c} \nabla c)-c{\mathcal {A}}_{c}], \end{aligned}$$

let us start by addressing first the diffusion part, $\nabla \cdot (D^{c} \nabla c)$, and only afterwards will formulate the computational approach for the adhesion part $\nabla \cdot c{\mathcal {A}}_{c}$.

#### Discretisation of the Diffusion Operator $\nabla \cdot (D^{c} \nabla c)$

Denoting for convenience the gradient flux of the cancer cells by

31 $$\begin{aligned} {\mathcal {F}}_{c,D} := D^{c} \nabla c, \end{aligned}$$

this diffusion part of the spatial operator $\nabla \cdot {\mathcal {F}}_{c}$ can therefore be rewritten as $\nabla \cdot (D^{c} \nabla c)=\nabla \cdot {\mathcal {F}}_{c,D}$, and to discretise this, we simply use a second-order central difference scheme with the usual 5-point stencil.

During the computations, we detect our expanding tumour domain $\varOmega (t_{0})$ via an indicator function denoted by ${\mathcal {I}}(\cdot , \cdot ) : X \times Y \rightarrow \{0,1\}$, with $X = Y = \{1,\ldots ,N\}$, and defined by

32 $$\begin{aligned} {\mathcal {I}}(i, j) := {\left\{ \begin{array}{ll} 1 &{} \text {if } (x_{i}, y_{j}) \in \varOmega (t_{0}),\\ 0 &{} \text {otherwise}, \end{array}\right. } \end{aligned}$$

which enables us to determine whether a spatial node is inside or outside the tumour domain. Further, we construct a similar indicator function for the boundary nodes. Hence, let us denote this by ${\mathcal {I}}_{B}(\cdot , \cdot ) : X \times Y \rightarrow \{0,1\}$ and define it as

33 $$\begin{aligned} {\mathcal {I}}_{B}(i, j) := {\left\{ \begin{array}{ll} 1 &{} \text {if } (x_{i}, y_{j}) \in \partial \varOmega (t_{0}),\\ 0 &{} \text {if } (x_{i}, y_{j}) \notin \partial \varOmega (t_{0}), \end{array}\right. } \end{aligned}$$

where $\partial \varOmega (t_{0})$ denotes the boundary of the domain $\varOmega (t_{0})$. These two functions allow us to split the macro-scale dynamics computation into two parts, i.e. strictly inside the tumour and on the tumour boundary. The motivation behind this is that, given the complexity of our multiscale algorithm (where *“on-the-fly”* rearrangements of micro-fibres are required), to reduce the computational time, we aim to apply some appropriately constructed universal discrete macro-scale numerical operators that would enable us to deal with the approximation of the right-hand side spatial operators at as many macro-nodes as possible at once, ultimately resulting in a macro-scale computational techniques that is faster than the one proposed in Shuttleworth and Trucu (2019). However, due to the continuously changing tumour domain $\varOmega (t)$ (resulting in an irregular tumour shape) and the presence of zero-flux boundary conditions for the tumour macro-dynamics, we cannot derive one global numerical approach for the dynamics at all the tumour macro-points, and rather we need to split the computation in these two parts (i.e. inside the tumour and tumour boundary) and deal with them separately.

For convenience, we also define the indicator function for the interior of $\varOmega (t_{0})$ by ${\mathcal {I}}_{I}(\cdot , \cdot ) = {\mathcal {I}}(\cdot , \cdot ) - {\mathcal {I}}_{B}(\cdot , \cdot )$ as

34 $$\begin{aligned} {\mathcal {I}}_{In}(i, j) := {\left\{ \begin{array}{ll} 1 &{} \text {if } (x_{i}, y_{j}) \in \varOmega (t_{0}) \text { and } (x_{i}, y_{j}) \notin \partial \varOmega (t_{0}),\\ 0 &{} \text {else}, \end{array}\right. } \end{aligned}$$

Then, using these two indicator functions (33) and (34), we also define two sets of points $\varLambda _{B}$ and $\varLambda _{In}$, containing the boundary and interior nodes, respectively,

35 $$\begin{aligned} \begin{aligned} \varLambda _{B}&{:=}\, \{(i,j) \in X \times Y | \; {\mathcal {I}}_{B}(i,j) = 1\},\\ \varLambda _{In}&{:=}\, \{(i,j) \in X \times Y | \; {\mathcal {I}}_{In}(i,j) = 1\}. \end{aligned} \end{aligned}$$

Since, aiming to reduce computational cost, our goal is to partition the problem into two main computational components, it is therefore indispensable to split appropriately the numerical approximation of the diffusion part of the spatial operator, namely of $\nabla \cdot {\mathcal {F}}_{c,D}$. For this, let us notice that at any spatial node $(x_{i},x_{j}) \in \varOmega (t_{0}) \subset Y$, the discretisation of $\nabla \cdot {\mathcal {F}}_{c,D}$ can actually be represented as follows

36 $$\begin{aligned} \nabla \cdot {\mathcal {F}}_{c,D}(i, j) = {\left\{ \begin{array}{ll} \nabla \cdot {\mathcal {F}}_{c,D}^{In}(i, j) &{} \text {if } (i,j) \in \varLambda _{I},\\ \nabla \cdot {\mathcal {F}}_{c,D}^{B}(i, j) &{} \text {if } (i,j) \in \varLambda _{B},\\ 0 &{} \text {else}. \end{array}\right. } \end{aligned}$$

Further, considering now the *three-row* matrices $\{T_{i}\}_{i=2,N-1}$

37 $$\begin{aligned} T_{i}:=[\underbrace{\mathbf{0 \dots \mathbf{0} }}_{i-2}, {\mathbf {I}}_{3}, \underbrace{\mathbf{0 \dots \mathbf{0} }}_{N-i-1}] \end{aligned}$$

where $\mathbf {0}$ represents a three-dimensional zero column vector and ${\mathbf {I}}_{3}$ is the $3\times 3$ identity matrix, for any $(i,j)\in \{2,\dots , N-1\}$, we extract the $3\times 3$ sub-matrices centred at (*i*, *j*) that contain the values at (*i*, *j*) and its corresponding eight neighbouring locations for the various constituents of the coupled dynamics. Thus, these submatrices are given by

38 $$\begin{aligned}{}[c^{n}_{i,j}] = T_{i} \; c^{n} \; T_{j}^{\intercal }, \qquad [(D^{c})^{n}_{i,j}] = T_{i} \; (D^{c})^{n} \; T_{j}^{\intercal }, \end{aligned}$$

where $c^{n}$ and $(D^{c})^{n}$ are the corresponding discretised values for the cell population and cell diffusion coefficient (that is dependent on macrophages and ECM fibres) at the mesh points $\{(x_{i}, y_{j})\}_{i,j=1\ldots N}$, at time step n. With these notations (detailed in (38)), we will be able to represent finite-difference approach for $\nabla \cdot {\mathcal {F}}_{c,D}$ in terms of matrix operations.

In this context, our scheme for spatial flux $\nabla \cdot {\mathcal {F}}_{c}(\cdot , \cdot )$ can be written down explicitly using central differences and midpoint approximations. Thus, for $\nabla \cdot {\mathcal {F}}_{c}^{In}(\cdot , \cdot )$, using the Hadamard product “$\circ$” (defined for completion in “Appendix D”, while for further details, we refer the reader to Horn and Johnson (1991)), $\forall (i,j) \in \varLambda _{In}$ and for any time step *n*, we have that

39 $$\begin{aligned} \begin{aligned} \big (\nabla \cdot {\mathcal {F}}_{c,D}^{In} \big )_{i,j}^n&= \frac{1}{\Delta x^2} \sum _{k=1}^{2} \Bigg [ \sum \Big ([(D^c)_{i,j}^n] \circ {\mathcal {K}}_{A}^{2k-1} \Big ) \cdot \sum \Big ([c_{i,j}^n] \circ {\mathcal {K}}_{F}^{k} \Big ) \\&\quad - \sum \Big ([(D^c)_{i,j}^n] \circ {\mathcal {K}}_{A}^{2k} \Big ) \cdot \sum \Big ([c_{i,j}^n] \circ {\mathcal {K}}_{B}^{k} \Big ) \Bigg ], \end{aligned} \end{aligned}$$

Here, $\sum$ denotes the Frobenius inner product (Golub and van Loan 2013), which, for completion, is also defined in “Appendix D”. Furthermore, the “${\mathcal {K}}$”s terms appearing in the above formula are the $3 \times 3$ discrete matrices induced by the finite-difference method at each $(i,j) \in \varLambda _{I}$. Specifically, for any $\,k=1\ldots 4$, each ${\mathcal {K}}_{A}^{k}$ describes the average between the centre point (*i*, *j*) and one of its neighbours and is given by the $3 \times 3$ associated matrices

40 $$\begin{aligned} {\mathcal {K}}_{A}^{1} \!=\! \begin{bmatrix} 0 &{} 0 &{} 0 \\ 0.5 &{} 0.5 &{} 0 \\ 0 &{} 0 &{} 0 \end{bmatrix}\!\!, \!\; {\mathcal {K}}_{A}^{2} \!=\! \begin{bmatrix} 0 &{} 0 &{} 0 \\ 0 &{} 0.5 &{} 0.5 \\ 0 &{} 0 &{} 0 \end{bmatrix}\!\!, \!\; {\mathcal {K}}_{A}^{3} \!=\! \begin{bmatrix} 0 &{} 0.5 &{} 0 \\ 0 &{} 0.5 &{} 0 \\ 0 &{} 0 &{} 0 \end{bmatrix}\!\!, \!\; {\mathcal {K}}_{A}^{4} \!=\! \begin{bmatrix} 0 &{} 0 &{} 0 \\ 0 &{} 0.5 &{} 0 \\ 0 &{} 0.5 &{} 0 \end{bmatrix}\!\!. \end{aligned}$$

Further, in (39) ${\mathcal {K}}_{F}^{k}$ and ${\mathcal {K}}_{B}^{k}$ denote the $3 \times 3$ matrices generated by the forward and backward differences, respectively. Hence, in both direction *i* (if $k=1$) and direction *j* (if $k=2$), these are given by:

41 $$\begin{aligned} {\mathcal {K}}_{F}^{1} \!=\! \begin{bmatrix} 0 &{} 0 &{} 0 \\ 1 &{} -1 &{} 0 \\ 0 &{} 0 &{} 0 \end{bmatrix}\!\!, \!\; {\mathcal {K}}_{B}^{1} \!=\! \begin{bmatrix} 0 &{} 0 &{} 0 \\ 0 &{} 1 &{} -1 \\ 0 &{} 0 &{} 0 \end{bmatrix}\!\!, \!\; {\mathcal {K}}_{F}^{2} \!=\! \begin{bmatrix} 0 &{} 1 &{} 0 \\ 0 &{} -1 &{} 0 \\ 0 &{} 0 &{} 0 \end{bmatrix}\!\!, \!\; {\mathcal {K}}_{B}^{2} \!=\! \begin{bmatrix} 0 &{} 0 &{} 0 \\ 0 &{} 1 &{} 0 \\ 0 &{} -1 &{} 0 \end{bmatrix}\!\!. \end{aligned}$$

Finally, we can observe that at the interior nodes of $\varOmega (t_{0})$, Eq. (39) can be equivalently expressed via discrete convolutions, and so we can write this as

42 $$\begin{aligned} \begin{aligned} \big (\nabla \cdot {\mathcal {F}}_{c,D}^{In} \big )^n&= \frac{1}{\Delta x^2} \sum _{k=1}^{2} \Bigg [ \Big ((D^c)^n *{\widetilde{{\mathcal {K}}}}_{A}^{2k-1} \Big ) \circ \Big (c^n *{\widetilde{{\mathcal {K}}}}_{F}^{k} \Big ) \\&\quad - \Big ((D^c)^n *{\widetilde{{\mathcal {K}}}}_{A}^{2k} \Big ) \circ \Big (c^n *{\widetilde{{\mathcal {K}}}}_{B}^{k} \Big ) \Bigg ], \end{aligned} \end{aligned}$$

where $*$ is the discrete convolution operator (Damelin and Miller 2011) for the discretised tumour dynamic constituents. Furthermore, due to the definition of discrete convolution, the ${\widetilde{{\mathcal {K}}}}$s used in (42) are appropriately derived from ${\mathcal {K}}$s (defined in (40) and (41)) and are given as

$$\begin{aligned} {\widetilde{{\mathcal {K}}}} := J_{3} {\mathcal {K}}J_{3}, \end{aligned}$$

where $J_{3}$ is the anti-diagonal identity matrix, i.e.

43 $$\begin{aligned} J_{3} = \begin{bmatrix} 0 &{} 0 &{} 1 \\ 0 &{} 1 &{} 0 \\ 1 &{} 0 &{} 0 \end{bmatrix}. \end{aligned}$$

However, for the boundary nodes, the discretisation formulated in (42) could not be applicable directly, and so for this we revisit (39) by taking into account now the zero-flux boundary conditions. These boundary conditions are accounted for through a new family of $3\times 3$ sub-matrices that are defined as follows:

44 $$\begin{aligned} \begin{aligned} \text{[ }c_{i,j}^{n}]^B&{:=}\, [c^{n}_{i,j}] \circ [{\mathcal {I}}_{i,j}^{n}] + (J_{3} [c^{n}_{i,j}] J_{3}) \circ \big ( \mathbb {1} -[{\mathcal {I}}_{i,j}^{n}] \big ), \\ [(D^{c})^{n}_{i,j}]^{B}&{:=}\, [(D^{c})^{n}_{i,j}] \circ [{\mathcal {I}}_{i,j}^{n}] + (J_{3} [(D^{c})^{n}_{i,j}] J_{3}) \circ \big ( \mathbb {1} -[{\mathcal {I}}_{i,j}^{n}] \big ), \end{aligned} \end{aligned}$$

where $\mathbb {1}$ is a $3 \times 3$ matrix of ones, $J_{3}$ is the anti-diagonal identity matrix defined in (43), while the $3 \times 3$ matrices $[{\mathcal {I}}_{i,j}^{n}]$ are given at each (*i*, *j*) by

45 $$\begin{aligned}{}[{\mathcal {I}}_{i,j}^{n}] := T_{i} \; {\mathcal {I}}^{n} \; T_{j}^{\intercal }. \end{aligned}$$

Therefore, using (44), we separate these boundary sub-matrices into two parts, i.e. a part that is inside the tumour and to another part that is outside the tumour (determined by the indicator function ${\mathcal {I}}$). Hence, in these $3 \times 3$ boundary sub-matrices, the distribution at the nodes that are inside the tumour is the same as the one defined in (38), but we appropriately provide values on the neighbouring nodes, captured by $[{\mathcal {I}}_{i,j}^{n}]$, outside the tumour so that we could implement the zero-flux boundary condition. Hence, at any time $t_{n}$, our scheme at any boundary node $(i,j) \in \varLambda _{B}$ is given by

46 $$\begin{aligned} \begin{aligned} \big (\nabla \cdot {\mathcal {F}}_{c,D}^{B} \big )_{i,j}^n&= \frac{1}{\Delta x^2} \sum _{k=1}^{2} \Bigg [ \sum \Big ([(D^c)_{i,j}^n]^{B} \circ {\mathcal {K}}_{A}^{2k-1} \Big ) \cdot \sum \Big ([c_{i,j}^n]^{B} \circ {\mathcal {K}}_{F}^{k} \Big ) \\&\quad - \sum \Big ([(D^c)_{i,j}^n]^{B} \circ {\mathcal {K}}_{A}^{2k} \Big ) \cdot \sum \Big ([c_{i,j}^n]^{B} \circ {\mathcal {K}}_{B}^{k} \Big ) \Bigg ], \end{aligned} \end{aligned}$$

where the “${\mathcal {K}}$” matrices involved here are the ones that we already defined in (40) and (41).

#### Discretisation of the *Adhesion* Operator $\nabla \cdot c{\mathcal {A}}_{c}$

Now we shift our attention to the cancer cell adhesion term $\nabla \cdot c {\mathcal {A}}_{c}$. Postponing for the moment, the spatial discretisation of the adhesion vector field ${\mathcal {A}}_{c}$ at time $t_{n}$, which we denote by ${\mathcal {A}}_{c}^{n}$, whose details will be discussed in Sect. G.2, in the following we will focus on addressing the overall discretisation of the adhesion operator $\nabla \cdot c{\mathcal {A}}_{c}$, for which we will propose a flux limiter approach.

Although also here we will split the discretised spatial tumour domain into two regions, these will be slightly different from the interior and boundary parts mentioned in the previous section. Further, since we aim here to use a flux limiter, such approach requires the usage of *“ghost” outside nodes* that are further away from the boundary nodes than their immediate neighbours (for instance, the ghost point $(i+2,j)$ if $(i,j) \in \varLambda _{B}$). Thus, we appropriately split $\varOmega (t_{0})$ into two regions that we refer to as the *“layer”* and *“inside”* parts.

Focusing first on the *inside* part, this is determined by the requirements of the flux limiter (detailed below), which, for calculations at position (*i*, *j*), demands availabilities for the values of the quantities involved on a region that includes the four neighbours (in both *i* and *j* directions) of the four neighbouring locations with respect to (*i*, *j*). Therefore, the *inside* part contains the discretised spatial region given by the macro-nodes of the domain $\varOmega (t_{0})$ that are at least two macro-nodes “distance” away from the boundary, hence this consists of all the points that are neither boundary points nor immediate neighbours of any boundary point. Thus, we can determine the *inside* part mathematically by considering the following indicator function denoted by ${\mathcal {I}}_{I}(\cdot , \cdot ) : X \times Y \rightarrow \{0,1\}$ and define by

47 $$\begin{aligned} {\mathcal {I}}_{I}(i, j) := {\left\{ \begin{array}{ll} 1 &{} \text {if } ({\mathcal {I}}*{\mathcal {K}}_{in})_{i,j} = 1,\\ 0 &{} \text {if } ({\mathcal {I}}*{\mathcal {K}}_{in})_{i,j} = 0, \end{array}\right. } \end{aligned}$$

where $*$ is the discrete convolution operator, ${\mathcal {I}}$ is defined in (32) and ${\mathcal {K}}_{in}$ is defined by

$$\begin{aligned} {\mathcal {K}}_{in} = \frac{1}{13} \begin{bmatrix} 0 &{} \quad 0 &{}\quad 1 &{}\quad 0 &{}\quad 0 \\ 0 &{}\quad 1 &{}\quad 1 &{}\quad 1 &{}\quad 0 \\ 1 &{}\quad 1 &{}\quad 1 &{}\quad 1 &{}\quad 1 \\ 0 &{}\quad 1 &{}\quad 1 &{}\quad 1 &{}\quad 0 \\ 0 &{}\quad 0 &{}\quad 1 &{}\quad 0 &{}\quad 0 \\ \end{bmatrix}, \end{aligned}$$

which is based on the stencil induced by the flux limiter scheme.

Turning now our attention to the *layer* part, the macro-nodes that are considered to be in this region of the discretised tumour domain $\varOmega (t_{0})$ are the ones that are either classified as boundary nodes or their immediate neighbours. The motivation behind this is again to treat nodes that use values at ghost/outside points differently than the ones that do not require such values during the approximation and would this way enable a reduction in the computational cost. Thus, the points in the *layer* region are simply given by the difference indicator function ${\mathcal {I}}_{L} := {\mathcal {I}}- {\mathcal {I}}_{I}$, i.e.,

48 $$\begin{aligned} {\mathcal {I}}_{L}(i, j) := {\left\{ \begin{array}{ll} 1 &{} \text {if } {\mathcal {I}}(i,j) - {\mathcal {I}}_{I}(i,j) = 1 ,\\ 0 &{} \text {if } {\mathcal {I}}(i,j) - {\mathcal {I}}_{I}(i,j) = 0. \end{array}\right. } \end{aligned}$$

Therefore, using (47) and (48), we obtain that the discretised *inside* and *layer* parts of $\varOmega (t_{0})$, denoted here by $\varLambda _{I}$ and $\varLambda _{L}$, are given by the preimages $\varLambda _{I}:={\mathcal {I}}_{I}^{-1}(\{1\})$ and $\varLambda _{L}:={\mathcal {I}}_{L}^{-1}(\{1\})$, respectively, i.e.

$$\begin{aligned} \begin{aligned} \varLambda _{I} := \{(i,j) \in X \times Y | \; {\mathcal {I}}_{I}(i,j) = 1\},\\ \varLambda _{L} := \{(i,j) \in X \times Y | \; {\mathcal {I}}_{L}(i,j) = 1\}. \end{aligned} \end{aligned}$$

Focusing now on computations on the *inside* region of $\varOmega (t_{0})$, at any time $t_{n}$ and any inside node $(i,j) \in \varLambda _{I}$, the cancer cell adhesion discretisation is defined by

49 $$\begin{aligned} \nabla \cdot (c {\mathcal {A}}_{c})_{i,j}^{n} := \dfrac{1}{\Delta x} \Big ( (H^{x})_{i,j}^{n} - (H^{x})_{i-1,j}^{n} \Big ) + \dfrac{1}{\Delta y} \Big ( (H^{y})_{i,j}^{n} - (H^{y})_{i,j-1}^{n} \Big ) \end{aligned}$$

where $H^{x}_{i,j}$ and $H^{y}_{i,j}$ are defined as

50 $$\begin{aligned} \begin{aligned} (H^{x})_{i,j}^{n}&{:=}\, \max \{0, \text {sgn}[({\mathcal {F}}_{c,{\mathcal {A}}}^{y})_{i,j}^{n}]\} (L_{x}^{+})_{i,j}^{n} + \min \{0, \text {sgn}[({\mathcal {F}}_{c,{\mathcal {A}}}^{x})_{i,j}^{n}]\} (L_{x}^{-})_{i,j}^{n}, \\ (H^{y})_{i,j}^{n}&{:=}\, \max \{0, \text {sgn}[({\mathcal {F}}_{c,{\mathcal {A}}}^{y})_{i,j}^{n}]\} (L_{y}^{+})_{i,j}^{n} + \min \{0, \text {sgn}[({\mathcal {F}}_{c,{\mathcal {A}}}^{y})_{i,j}^{n}]\} (L_{y}^{-})_{i,j}^{n}. \end{aligned} \end{aligned}$$

Here, for a compact notation, $({\mathcal {F}}_{c,{\mathcal {A}}}^{x})_{i,j}^{n}$ and $({\mathcal {F}}_{c,{\mathcal {A}}}^{y})_{i,j}^{n}$ denote the cancer cell adhesion fluxes and they are given by

51 $$\begin{aligned} \begin{aligned} ({\mathcal {F}}_{c,{\mathcal {A}}}^{x})_{i,j}^{n}&{:=}\, c_{i,j}^n {\mathcal {A}}_{c}^{x}(t_{n}, \mathbf{u }_{i,j}^{n}, (\theta _{f})_{i,j}^{n}), \\ ({\mathcal {F}}_{c,{\mathcal {A}}}^{y})_{i,j}^{n}&{:=}\, c_{i,j}^n {\mathcal {A}}_{c}^{y}(t_{n}, \mathbf{u }_{i,j}^{n}, (\theta _{f})_{i,j}^{n}), \end{aligned} \end{aligned}$$

where $\mathbf{u }_{i,j}^{n} = (c_{i,j}^{n}, F_{i,j}^{n}, l_{i,j}^{n}, M_{i,j}^{n})^{T}$ (defined in (1)) with the matrices $c^{n}$, $F^{n}$, $l^{n}$, $M^{n}$ and $(\theta _{f})^{n}$ represent the discrete values at the grid points of the cancer cells, fibres ECM, non-fibres ECM, M2 TAMs, and oriented ECM fibres, respectively. Also, in (51) ${\mathcal {A}}_{c}^{x}$ and ${\mathcal {A}}_{c}^{y}$ denote the *x* and *y* components of the adhesion vector field ${\mathcal {A}}_{c}$, respectively.

Furthermore, in Eq. (50), the terms $(L_{x}^{+})_{i,j}^{n}$, $(L_{x}^{-})_{i,j}^{n}$, $(L_{y}^{+})_{i,j}^{n}$ and $(L_{y}^{-})_{i,j}^{n}$ are usually referred to as *state interpolants*, and to construct these, we involve the so-called *limiter function* $\phi (r)$, which ultimately enables us to use second-order approximation when the solution is smooth and a first-order approximation near sharp gradients. Hence, these state interpolants at time $t_{n}$ and any inside node $(i,j) \in \varLambda _{I}$ are defined by

52 $$\begin{aligned} \begin{aligned} (L_{x}^{+})_{i,j}^{n}&{:=}\, ({\mathcal {F}}_{c}^{x})_{i,j}^{n} + \dfrac{1}{2} \phi (r^{x}_{i,j}) \Big ( ({\mathcal {F}}_{c}^{x})_{i,j}^{n} - ({\mathcal {F}}_{c}^{x})_{i-1,j}^{n} \Big ), \\ (L_{x}^{-})_{i,j}^{n}&{:=}\, ({\mathcal {F}}_{c}^{x})_{i+1,j}^{n} + \dfrac{1}{2} \phi \Bigg ( \dfrac{1}{r^{x}_{i+1,j}} \Bigg ) \Big ( ({\mathcal {F}}_{c}^{x})_{i+1,j}^{n} - ({\mathcal {F}}_{c}^{x})_{i+2,j}^{n} \Big ), \\ (L_{y}^{+})_{i,j}^{n}&{:=}\, ({\mathcal {F}}_{c}^{y})_{i,j}^{n} + \dfrac{1}{2} \phi (r^{y}_{i,j}) \Big ( ({\mathcal {F}}_{c}^{y})_{i,j}^{n} - ({\mathcal {F}}_{c}^{y})_{i,j-1}^{n} \Big ), \\ (L_{y}^{-})_{i,j}^{n}&{:=}\, ({\mathcal {F}}_{c}^{y})_{i,j+1}^{n} + \dfrac{1}{2} \phi \Bigg ( \dfrac{1}{r^{y}_{i,j+1}} \Bigg ) \Big ( ({\mathcal {F}}_{c}^{y})_{i,j+1}^{n} - ({\mathcal {F}}_{c}^{y})_{i,j+2}^{n} \Big ), \end{aligned} \end{aligned}$$

where $r^{x}_{i,j}$ and $r^{y}_{i,j}$ are the *smoothness monitor functions* in *x*- and *y*-directions, respectively, i.e., they are given by

53a $$\begin{aligned} r^{x}_{i,j} := {\left\{ \begin{array}{ll} \dfrac{({\mathcal {F}}_{c}^{x})_{i+1,j}^{n} - ({\mathcal {F}}_{c}^{x})_{i,j}^{n}}{({\mathcal {F}}_{c}^{x})_{i,j}^{n} - ({\mathcal {F}}_{c}^{x})_{i-1,j}^{n}} &{} \begin{aligned} &{} \text {if } ({\mathcal {F}}_{c}^{x})_{i,j}^{n} \ne ({\mathcal {F}}_{c}^{x})_{i-1,j}^{n} \\ &{} \text {and } ({\mathcal {F}}_{c}^{x})_{i+1,j}^{n} \ne ({\mathcal {F}}_{c}^{x})_{i,j}^{n}, \end{aligned} \\ 0 &{} \text {otherwise}, \end{array}\right. } \end{aligned}$$

53b $$\begin{aligned} r^{y}_{i,j} := {\left\{ \begin{array}{ll} \dfrac{({\mathcal {F}}_{c}^{y})_{i,j+1}^{n} - ({\mathcal {F}}_{c}^{y})_{i,j}^{n}}{({\mathcal {F}}_{c}^{y})_{i,j}^{n} - ({\mathcal {F}}_{c}^{y})_{i,j-1}^{n}} &{} \begin{aligned} &{} \text {if } ({\mathcal {F}}_{c}^{y})_{i,j}^{n} \ne ({\mathcal {F}}_{c}^{y})_{i,j-1}^{n} \\ &{} \text {and } ({\mathcal {F}}_{c}^{y})_{i,j+1}^{n} \ne ({\mathcal {F}}_{c}^{y})_{i,j}^{n}, \end{aligned} \\ 0 &{} \text {otherwise}. \end{array}\right. } \end{aligned}$$

In all of our numerical simulations (presented Sect. 3), we use the so-called *UMIST limiter function* (Lien and Leschziner 1994) (although there are more limiter functions available (Kuzmin 2006)) that is given by

$$\begin{aligned} \phi (r) := \max [0, \min (2r, 0.25+0.75r, 0.75 + 0.25r, 2)], \end{aligned}$$

which completes the flux limiter part of the scheme for any inside node $(i,j) \in \varLambda _{I}$ at time $t_{n}$.

Finally, we turn our attention to the computations on the *layer* part $\varLambda _{L}$ of the discretised tumour domain $\varOmega (t_{0})$. To approximate the cancer cell adhesion term $\nabla \cdot c {\mathcal {A}}_{c}$ in this region, we use a first-order upwind scheme, which we obtain by choosing $\phi \equiv 0$ in (52). Hence, with $\phi \equiv 0$, we can now use directly the method derived in (49)–(53) for any node that is a layer point, but not a boundary point, i.e., at all spatial nodes $(i,j) \in \varLambda _{L} \setminus \varLambda _{B}$. This leaves us with only the boundary nodes where we still need to address the computation. Since at these points we also aim to use the unwinding scheme, we need to appropriately approximate the value of any ghost point. To that end, we involve a first-order approximation of the cancer cell zero-flux boundary condition which then enables us to appropriately estimate the values at the required ghost/outside point. Finally, this allows us to use the method derived in (49)–(53), for any boundary node $(i,j) \in \varLambda _{B}$ at any time step *n*, with $\phi \equiv 0$. Therefore, we have fully defined our numerical scheme for the spatial transport of the cancer cell population i.e. for $\nabla \cdot {\mathcal {F}}_{c}$. To avoid repetition, we did not include here also the details of the derivation of the numerical scheme for the M2 TAM macrophages, as, due to the similarity in the structures of the spatial operators (involved in the transport of M2 TAMs), the numerical treatment is identical to the one outlined for the cancer cell equation. Hence, the scheme derived in this section is straightforward to implement for the M2 TAMs as well, or in fact for any other population with the same spatial operators and so we do not pay special attention for this procedure.

### Adhesion Terms

Focusing now our attention to the sensing region $x+{\mathbf {B}}(0,R)$, where the cell-adhesion processes cell–fibres ECM adhesion and cell–non-fibres ECM adhesion, as well as cell–cell self-adhesion and cell–cell adhesion for both cancer cells and M2 TAMs macrophages populations (modelled in (5) and (12)) are exercised, we adopt the partitioning of this region from Shuttleworth and Trucu (2019) as illustrated in Fig. 14. Furthermore, as the computational procedure is identical for both adhesion operators ${\mathcal {A}}_{c}$ (that is associated with cancer cell) and $A_{M}$ (that is associated with M2 TAMs macrophages), let us detail this approach simultaneously, i.e. for the adhesion operator ${\mathcal {A}}\in \{{\mathcal {A}}_{c},{\mathcal {A}}_{M}\}$.

Thus, let us denote the number of annulus sectors in this partitioning by $N_{s}$, and since this determined by the intersection of each of the *s* annuli centred at *x* with a number of uniformly distributed sectors $2^{m+(k-1)}$ (which is dyadic-ally increasing as *k* progresses from the first and inner most annulus to that last and biggest one), $k\in \{1,\dots , s\}$, we have that

$$\begin{aligned} N_{s} := \sum _{k=1}^{s} 2^{m+(k-1)}, \end{aligned}$$

Then, denoting these annulus sectors by $S_{\nu }$, with $\nu = 1{,\dots ,}N_{s}$, this enables us to approximate the adhesion integral (5) the integral of the step function associated with these sectors, whose value on each $S_{\nu }$ is appropriately constructed as a linear combination of the mean values on $S_{\nu }$ of the cancer cell populations, M2 TAMs macrophages and ECM components involved in the adhesion processes described by (5) and (12).

Hence, to calculate these mean values, on each $S_{\nu }$, we first need to integrate the cancer and M2 TAM macrophages populations, the non-fibres ECM, the fibres ECM as well as the fibres orientation on $S_{\nu }$ . For this, we denote the off-grid barycentres of each annulus sector by $b_{S_{\nu }}$ and observe first that each $b_{S_{\nu }}$ belongs to the rectangle defined by its four immediately surrounding on-grid macro-mesh neighbouring nodes $\{y_{b_{S_{\nu }}}^i\}_{i=1,4}$, as illustrated in Fig. 14c, namely

54 $$\begin{aligned} b_{S_{\nu }} \in [y_{b_{S_{\nu }}}^1, y_{b_{S_{\nu }}}^3] \times [y_{b_{S_{\nu }}}^2, y_{b_{S_{\nu }}}^4], \end{aligned}$$

Then, we use bilinear shape functions to obtain the values of cancer population, M2 TAMs macrophages, the non-fibres ECM, the fibres ECM as well as the fibres orientation on $S_{\nu }$ at $b_{S_{\nu }}$ as a convex combination with uniquely determined weights $\beta ^{k}_{\nu }$, $k=1{,\dots ,}4$ of the values these quantities carry at the neighbouring on-grid points $\{y_{b_{S_{\nu }}}^i\}_{i=1,4}$.

Finally, since we seek to use again convolutions for our calculation, $N_{s}$ different matrices are constructed (one for each barycentre) that we denote here by ${\mathcal {K}}_{{\mathcal {A}}}^{S_{\nu }}$. For this, considering an arbitrary annulus *k* and a sector *j* that defines $S_{\nu }$, the associated ${\mathcal {K}}_{{\mathcal {A}}}^{S_{\nu }}$ is given by

$$\begin{aligned} {\mathcal {K}}_{{\mathcal {A}}}^{S_{\nu }} = \begin{bmatrix} 0 &{} \dots &{} \dots &{} \dots &{} \dots &{} 0 \\ \vdots &{} &{}\ddots &{} &{} \\ 0 &{} \dots &{} \beta _{\nu }^{1} &{} \beta _{\nu }^{3} &{} \dots &{} 0 \\ 0 &{} \dots &{} \beta _{\nu }^{2} &{} \beta _{\nu }^{4} &{} \dots &{} 0 \\ \vdots &{} &{} &{} \ddots &{} \\ 0 &{} \dots &{} \dots &{} \dots &{} \dots &{} 0 \\ \end{bmatrix}, \end{aligned}$$

which is a $P\times P$-matrix (with $P:= (\lceil 2 R / \Delta x \rceil + 1)$) that corresponds to the on-grid minimal squared regions of macro-mesh points $\{y_{x}^{i}\}_{i=1, P^{2}}$ that cover the sensing region ${\mathbf {B}}(x,R)$, and that is nonzero only on the four positions corresponding to the barycentre $b_{S_{\nu }}$ neighbouring locations where this is given by the weights $\beta ^{k}_{\nu }$, $k=1{,\dots ,}4$.

Therefore, at each instance of time $t_{n}$, the approximation of the adhesion integral ${\mathcal {A}}\in \{{\mathcal {A}}_{c},{\mathcal {A}}_{M}\}$ is given by

$$\begin{aligned} {\mathcal {A}}^{n} = \sum _{\nu = 1}^{N_{s}} {\mathcal {A}}_{S_{\nu }}^{n}, \end{aligned}$$

where the expression of ${\mathcal {A}}_{S_{\nu }}^{n}$ for each of the two adhesion operators in $\{{\mathcal {A}}_{c},{\mathcal {A}}_{M}\}$ is as follows:

- for ${\mathcal {A}}:={\mathcal {A}}_{c}$, we have
  $$\begin{aligned} \begin{aligned} ({\mathcal {A}}_{c})_{S_{\nu }}^{n}&= \frac{K(b_{S_{\nu }})}{R} \bigg [ \Big ( n_{\nu } {\widetilde{{\mathcal {K}}}}_{{\mathcal {A}}}^{S_{\nu }} \Big ) *\Big ( {\mathbf {S}}_{cc} c^{n} + {\mathbf {S}}_{cl} l^{n} + {\mathbf {S}}_{cM} M^{n} \Big ) \\&\quad + \Big ( {\widehat{n}}_{\nu } {\widetilde{{\mathcal {K}}}}_{{\mathcal {A}}}^{S_{\nu }} \Big ) *\Big ( {\mathbf {S}}_{cF} F^{n} \Big ) \bigg ] \bigg [ 1-\rho \Big ( \mathbf{u }_{b_{S_{\nu }}}^{n} \Big ) \bigg ]^{+}. \end{aligned} \end{aligned}$$
- for ${\mathcal {A}}:={\mathcal {A}}_{M}$, we have
  $$\begin{aligned} \begin{aligned} ({\mathcal {A}}_{M})_{S_{\nu }}^{n}&= \frac{K(b_{S_{\nu }})}{R} \bigg [ \Big ( n_{\nu } {\widetilde{{\mathcal {K}}}}_{{\mathcal {A}}}^{S_{\nu }} \Big ) *\Big ( {\mathbf {S}}_{MM} M^{n} + {\mathbf {S}}_{Mc} c^{n} \Big ) \\&\quad + \Big ( {\widehat{n}}_{\nu } {\widetilde{{\mathcal {K}}}}_{{\mathcal {A}}}^{S_{\nu }} \Big ) *\Big ( {\mathbf {S}}_\mathrm{MF} F^{n} \Big ) \bigg ] \bigg [ 1-\rho \Big ( \mathbf{u }_{b_{S_{\nu }}}^{n} \Big ) \bigg ]^{+}. \end{aligned} \end{aligned}$$

with ${\widetilde{{\mathcal {K}}}}_{{\mathcal {A}}}^{S_{\nu }}$ being the flipped matrix associated with ${\mathcal {K}}_{{\mathcal {A}}}^{S_{\nu }}$ obtained with the help of the $J_{3}$ anti-diagonal identity matrix defined in (43), i.e.

$$\begin{aligned} {\widetilde{{\mathcal {K}}}}_{{\mathcal {A}}}^{S_{\nu }}:=J_{3} {\mathcal {K}}_{{\mathcal {A}}}^{S_{\nu }} J_{3}. \end{aligned}$$

### Boundary Micro-Scale Dynamics

To calculate the source term $h(\cdot , \cdot )$ defined in (19) which is used in the MDE micro-scale equation (20), we need to integrate the cancer and M2 TAMs densities. For any such integral, we can use discrete convolutions with an appropriately chosen matrix ${\mathcal {K}}_{I}$ that will be detailed below. As before, we split the source into two parts

$$\begin{aligned} h(i,j) = {\left\{ \begin{array}{ll} h_{I}(i,j) &{} \text {if } (i,j) \in \varLambda _{In} \cup \varLambda _{B},\\ 0 &{} \text {else}. \end{array}\right. } \end{aligned}$$

Hence, at time $t_{n}$ the source inside the tumour domain is given by

55 $$\begin{aligned} h_{I}^{n} = 4 \gamma ^2 \dfrac{(N_{f} - 1)^{2}}{{\mathcal {I}}*\mathbb {1}} \circ \Big [ \big ( \alpha _{c} c^{n} + \alpha _{M} M^{n} \big ) *{\mathcal {K}}_{I} \Big ], \end{aligned}$$

where ${\mathcal {I}}$ was defined in (32), $\mathbb {1}$ is a matrix of ones with size being the same as ${\mathcal {K}}_{I}$ that is a $[2\gamma / \Delta x + 1] \times [2\gamma / \Delta x + 1]$ matrix with $\gamma$ defined in (19). Equation (55) is simply the measure of the boundary domain $\epsilon Y$ multiplied by the ratio of the nodes that lie inside the tumour times the integral. For our calculations, we construct ${\mathcal {K}}_{I}$ via the trapezoidal method, and so this is given by

$$\begin{aligned} {\mathcal {K}}_{I} = \begin{bmatrix} 1 &{} 2 &{} \dots &{} 2 &{} 1 \\ 2 &{} 4 &{} \dots &{} 4 &{} 2 \\ \vdots &{} \vdots &{}\ddots &{} \vdots &{} \vdots \\ 2 &{} 4 &{} \dots &{} 4 &{} 2 \\ 1 &{} 2 &{} \dots &{} 2 &{} 1 \\ \end{bmatrix}. \end{aligned}$$

At this point, we need to note that besides the usual error that the trapezoidal method gives due to the discontinuity we have in $B(y, \gamma ) \cap \varOmega (t)$ using a universal matrix for the integration has its drawbacks. This is because when we use the same matrix ${\mathcal {K}}_{I}$ for each $B(y, \gamma ) \cap \varOmega (t)$, then essentially we ignore that the function is discontinuous, resulting in an increase in the coefficients of the boundary nodes. On the other hand, by constructing unique kernels, we also run into difficulties in some cases; therefore, a universal matrix ${\mathcal {K}}_{I}$ was chosen to calculate the integral.

Since both cancer and macrophage densities are on the macro-scale, and we need to solve the MDE equation defined in (20) on the micro-scale, the source needs to be approximated on each micro-point *y*. To do this, we adopt the technique described in Trucu et al. (2013), i.e. involving bilinear shape functions on a square micro-mesh. Finally, due to the simplicity of the equation, we involve the Method of Lines, where the time-marching is carried out via the backward Euler method, while the space discretisation is based on central differences.
